# Rigorous monitoring of a large-scale marine stock enhancement program demonstrates the need for comprehensive management of fisheries and nursery habitat

**DOI:** 10.1038/s41598-019-39050-3

**Published:** 2019-03-27

**Authors:** Shuichi Kitada, Kaori Nakajima, Katsuyuki Hamasaki, Hirotoshi Shishidou, Robin S. Waples, Hirohisa Kishino

**Affiliations:** 1 Tokyo University of Marine Science and Technology, Tokyo, 108-8477 Japan; 2 Kagoshima Prefectural Fisheries Technology and Development Center, Kagoshima, 891-0315 Japan; 3 Northwest Fisheries Science Center, NOAA, Seattle, WA 98112 USA; 4 Graduate School of Agriculture and Life Sciences, The University of Tokyo, Tokyo, 113-8657 Japan

## Abstract

Release of captively-bred individuals into the wild is one of the most popular tools in fisheries, forestry, and wildlife management, and introgression of hatchery-reared animals into wild populations is of global concern. However, research and monitoring of impacts on wild populations are generally lacking, and the benefit of hatcheries for long-term fisheries and conservation goals is unclear. Using spatio-temporal genetic monitoring and a four-dacade time series of catch data, we quantified the effects on the size and genetic diversity of wild populations of one of the world’s largest marine stock enhancement programs–the red sea bream (*Pagrus major*) in Kagoshima Bay, Japan. Our analyses found that the stock enhancement program reduced genetic diversity of the population, but the genetic effect diminished with increased size of the wild population. Increases to the seaweed communities and reduced fishing efforts were the primary factors associated with the wild population recovery; effects of aquaculture were much smaller. Our results represent crucial evidence that hatcheries for enhancement and conservation of populations cannot be successful over the long term unless sufficient efforts are also made to reduce harvest rates and rehabilitate natural habitats.

## Introduction

Hatchery release is one of the most popular tools in fisheries, forestry, and wildlife management^1^, and it has become a standard conservation tool for Atlantic and Pacific salmon stocks listed under the Endangered Species Act in the USA and at a risk in Canada^2^. Huge numbers (>2.6 × 10^10^) of juveniles of 180 marine species are released into the wild every year in more than 20 countries, but in most cases monitoring efforts are insufficient to properly assess program effectiveness^3^. Research and monitoring of possible impacts on wild populations are generally lacking^1^. Although the ability of artificial propagation to maintain populations in the short term is well documented^3,4^, the benefit of hatcheries for long-term fisheries and conservation objectives remains unclear^4,5^. The most serious concern is genetic impacts of hatchery-reared animals on wild populations, which has long been discussed for salmon and marine species^6–10^. Modern parentage assignment studies provide evidence that the relative reproductive success (RRS) of hatchery-reared steelhead trout^11^, Chinook and coho salmon^12^ and farmed Atlantic salmon^13,14^ can be only half or less that of wild fish. Marine aquaculture is rapidly growing in Asia, the Mediterranean, and other areas in the world^3^, and genetic effects of huge escapements from fish farms have become a real concern^15^. Introgression of farmed Atlantic salmon into 62 wild Norwegian populations of Atlantic salmon was reported at ~6% on average^15–17^, with varying effects on size and age at maturity^18^ and other phenotypic and demographic characteristics^19,20^. More recently, genetic impacts of farm escapees have been reported for marine fishes^21–24^. A systematic review on marine stock enhancement and sea ranching highlighted evidence for substantial gene flow from hatcheries, reduction in genetic diversity in stocked populations, and changes in population structure and life history traits. However, empirical evidence for fitness reduction in stocked and/or farm-escaped populations is lacking, which might be attributed to intrinsic difficulty in collecting the appropriate data^3^. A rigorous evaluation would need simultaneous monitoring data for time series of population abundance, incorporated with stocking effects and genetic diversity. However, such data are largely not available due to the difficulty of long-term monitoring.

Using a spatio-temporal genetic monitoring and four-dacade time series of catch data, we quantified the effects on the size and genetic diversity of wild populations of one of the world’s largest marine stock enhancement programs–the red sea bream (*Pagrus major*) in Kagoshima Bay, Japan. Red sea bream is a commercially important, demersal fish widely distributed along the Japanese coasts. It is an iconic species for the Japan marine stock enhancement program, with ~9 million juveniles released every year^3^. Red sea bream mature at 3-4 years^25^ and spawn during March-May in western Japan. Pelagic larvae are transported to coastal areas and/or bays by tidal currents^26^. They grow into pelagic juveniles of 10 mm total length about 30 days after hatching and settle on coastal areas or inner bays when juveniles become demersal^26^. In this demersal stage, the juveniles mainly feed on copepods, which are abundant near the bottom and in seaweed communities^26^. Density gradients of copepods are the driver for early juveniles to move into their nursery grounds in shallow waters, where gammaridean amphipods, the most important foods for demersal juveniles, are abundant^26^. When juveniles grow to ~7 cm, young red sea bream begin to leave nursery grounds and move to sallow sandy bottoms. The most critical habitat could be seaweed beds for red sea bream throughout the life cycle, because its habitat complexity reduces vulnerability of juvenile and may determines the year-class strength^27^. We also quantify the effect of the KB hatchery releases and farm escapees in the top production area on genetic diversity of wild population, based on our spatio-temporal monitoring data for red sea bream. Finally, we infer the effect of captive breeding on fitness of stocked populations by analyzing published genotype data of the red sea bream in Japan and revisiting the results of previous studies.

## Results

### Stocking effect

**Figure 1:**
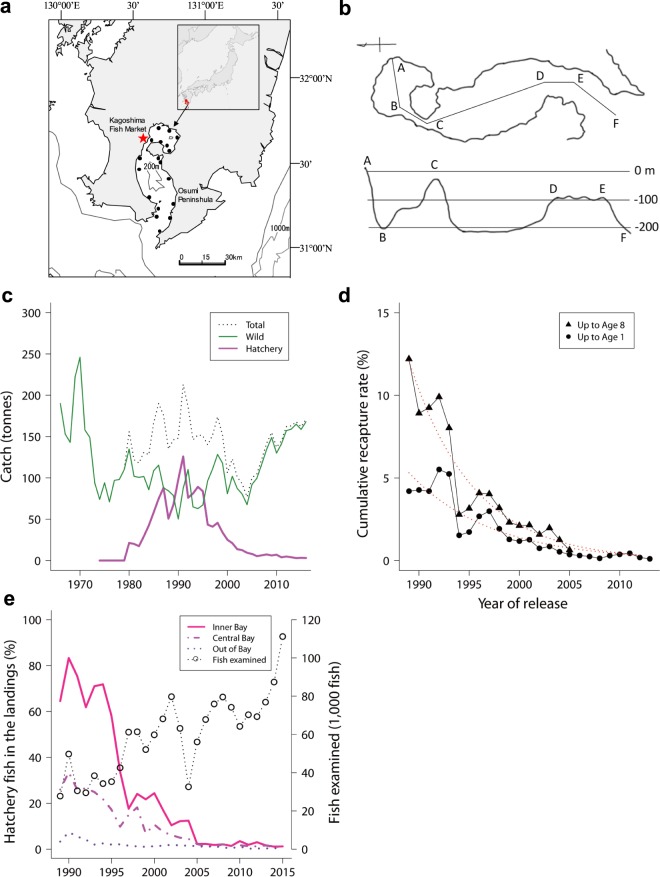
Red seabream stock enhancement in Kagoshima Bay (KB). (**a**) Release sites, and the Kagoshima City Fish Market (red star). (**b**) Plane figure and cross-section of KB. (**c**) Commercial landings in KB for total, wild, and hatchery released fish (1966–2016). (**d**) Cumulative recapture rates up to age 1 and age 8, corrected by fishing effort with fitted curves [*y* = 6.0143 exp(−0.1222*x*), *y* = 14.1090 exp(−0.1565*x*)]. (**e**) Proportion of hatchery fish in the KB commercial landings and sample sizes.

KB is a semi-closed bay of 1,129 km^2^ with an 8.7 km wide mouth (Fig. 1a,b). The total coastline is 330 km and ~60% has been artificially modified, most often in 1970s. The inner bay is a 250-km^2^ gigantic volcanic caldera shaped like a deep pond with maximum depth of 206 m, and the central bay is 580 km^2^ with maximum depth of 237 m. Habitat has been secured for each life stage of red sea bream and most of released juveniles could remain in the bay throughout the life cycle. Therefore, the hatchery release program expected intra- and inter-generational stoking effects in the bay. In KB, ~27 million hatchery-reared red sea bream (6–7 cm in total length) have been released since 1974 to achieve the goal of recovering the depleted commercial catch. The annual number of juveniles released ranged from 55 to 1,297 thousand and the average ± standard deviation (s.d.) was 636 ± 354 thousand. Red sea bream are caught by pole and line, gochi-nets (surrounding seine), gill nets, and long lines in KB. More than 80% of red sea bream caught in KB are landed at the Kagoshima City Fish Market (KCFM). During 1989–2015, surveys of Kagoshima Prefectural Fisheries Technology and Development Center (KPFTDC) examined ~1.6 million red sea bream to identify hatchery fish caught in KB and landed at fish markets (see Supplementary Information). We organized various datasets for monitoring stocking effects, including environmental data to quantify the effect of the KB red seabream stock enhancement program. Age composition and mean body weights of hatchery and wild fish by age were estimated based on the age–weight key, where ages were determined by reading otolith annuli. Age classes were one to eight, with the last being a “plus” class that included fish older than eight. The total numbers of landings were estimated for hatchery and wild fish by age classes based on commercial landings.

Catch of hatchery fish increased after the start of the program and reached 126 tonnes in 1991, but then consistently decreased and by 2016 had dropped to 3 tonnes (Fig. 1c). Cumulative recapture rates for up to 1-year-old and for up to 8-year-old red sea bream in KB, corrected by fishing effort, have decreased 12.2 ± 1.8% (standard error, s.e.m.) and 15.6 ± 1.7% per year, respectively, since 1994 (Fig. 1d). The recapture rates were similar at 13.2 ± 1.8% and 17.1 ± 1.8% without correction of fishing effort, respectively. We used corrected recapture rates in the subsequent analysis. Significant exponential decline rates were found for both 1-year-old and 8-year-old fish (*t* = 7.135, *P* = 2.88 × 10^−7^; *t* = 9.023, *P* = 1.90 × 10^−7^), but there was no significant difference between them, with the average of 13.9 ± 1.2% (*z* = 1.404, *P* = 0.0801). Since 1989, a total of 1,598,560 red sea bream landed on fish markets have been checked for the deformity of the internostril epidermis (DIE, see Supplementary Information) (Fig. 1e). The estimated proportion of hatchery fish (*P*_h_) in the landings in the inner KB (IKB), central KB, and outside the bay were highest in 1990 at 83.3%, 33.5%, and 7.4%; however, *P*_h_ decreased consistently after 1991, and hatchery fish currently contribute only ~1.0% to KB landings.

**Figure 2:**
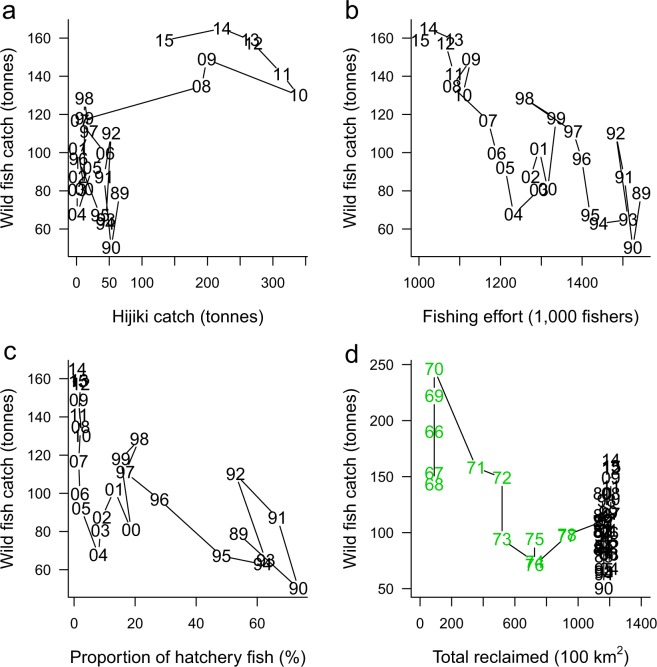
Wild red sea bream catches vs. environmental factors in KB. (**a**) Hijiki catch (edible brown alga, *Sargassum fusiforme*) (*r* = 0.71, *t* = 4.9916, *P* = 3.81 × 10^−5^, df = 25). (**b**) Fishing efforts (*r* = −0.81, *t* = −6.9499, *P* = 2.76 × 10^−7^, df = 25). (**c**) Proportion of hatchery fish (*P*_h_) (*r* = −0.68, *t* = −4.6129, *P* = 1.02 × 10^−4^, df = 25). (**d**) Total area of reclaimed land during 1966–1978 before hatchery-reared fish were landed as indicated in green (*r* = −0.82, *t* = 4.7799, *P* = 0.00057, df = 11). The numbers in each panel show years. (**a**–**c**), 1989–2015 and (**d**), 1966–2015.

In contrast to declining catch of hatchery fish, the catch of wild fish began to increase after 1991 and in 2016 reached the maximum post- release program of 168 tonnes (Fig. 1c). This indicated substantial increase in population size because fishing effort has linearly decreased for the past four decades (see Supplementary Information). The catch of wild fish was positively correlated with the harvest of Hijiki (brown alga, *Sargassum fusiforme*) in KB during the increasing period of 1989–2015 (Fig. 2a). In contrast, fishing effort and *P*_h_ were negatively correlated with the catch of wild fish (Fig. 2b,c). A negative correlation was also found for accumulated reclaimed land before released fish were landed during 1966–1978, suggesting that the reclamation of coastal areas substantially reduced wild red sea bream in 1970s (Fig. 2d). The variable selection in the step-wise linear regression analysis of the wild fish catch during the increasing period (1989–2015) excluded the SST (sea surface temperature) and *P*_h_ from the explanatory variables (Table 1). Instead, 69% of the variation in the increased wild catch was explained by a decrease in the fishing efforts and an increase in the Hijiki catch [*R*^2^ = 0.69, *F* = 30.5, *P* = 2.61 × 10^−7^, df = (2, 24)]. The AIC value for the selected model was 160.92 and that of the full model was 164.77, a result that is consistent with the recent increase in the community size of Hijiki in KB (see Supplementary Information).

**Table 1:** Factors associated with increased wild red seabream catches during 1989–2015 in Kagoshima Bay, Japan: the estimates of the linear regression with variable selection.

	Estimate	s.e.	*t*	*P*
Intercept	254.4811	39.0647	6.514	9.76 × 10^−7^
Hijiki catch	0.0982	0.0441	2.225	0.0357
Fishing effort	−0.1226	0.0285	−4.303	0.00024

### Genetic effect

**Figure 3:**
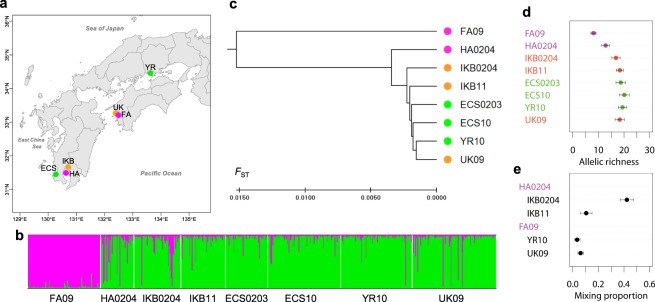
Genetic monitoring of red seabream. (**a**) Sampling sites: green = wild fish; orange = wild fish in impacted areas; magenta = hatchery and farmed fish (see Supplementary Table S1). (**b**) STRUCTURE bar plot assuming *K* = 2 populations). Numbers after sampling sites show years of sampling. (**c**) EB *F*_ST_ UPGMA dendrogram. (**d**) Allelic richness. (**e**) Mixing proportions of hatchery fish in IKB, and those of farmed fish in YR and UK.

We sequenced 493 red sea bream for the mtDNA-control region (CR) and 238 different haplotypes were obtained. We also genotyped 642 individuals collected during 2002 and 2011 (497 wild, 45 hatchery-recaptured, and 100 farmed fish) at nine microsatellite loci, but we only used the genotypes of five loci successfully genotyped (Fig. 3a, Supplementary Table S1). Hatchery, farmed and wild fish in IKB had smaller numbers of haplotypes and alleles (Supplementary Figs S1 and S2). The Bayesian clustering analysis suggested two putative original populations (*K* = 2), which are mostly explained by genetic differentiation between farmed and the other samples (Fig. 3b). Hatchery fish recaptured in KB (HA0204) had relatively larger farmed origins and were genetically more similar to wild fish in IKB during 2002 and 2004 (IKB0204), and different from other wild samples, whereas wild fish collected in IKB in 2011 (IKB11) was closer to other wild samples. The wild fish sample (UK09) collected from the Uwakai Sea (the most intensive aquaculture area, where ~35 thousand tons was annually produced, ~57% of all of Japan), had slightly larger farmed origins than in Yorishima (YR10) in the Seto Inland Sea, where no aquaculture or hatchery release has been in operation (see Supplementary Information). The posterior mean of the empirical Bayes pairwise *F*_ST_ for high gene flow species (EB*F*_ST_) was 0.01122 ± 0.01140 (s.d.), and the UPGMA dendrogram described the population structure consistent with the Bayesian clustering analysis (Fig. 3c). The allelic richness of farmed, hatchery, and IKB0204 were lower than other wild populations, but that of IKB11 recovered to the level of wild populations (Fig. 3d, Supplementary Table S2). The results from mtDNA-CR were consistent with those from microsatellite markers (Supplementary Fig. S3). The test of homogeneity for the haplotype and allele frequencies supported the above results (Supplementary Tables S3 and S4). The mixing proportions of hatchery fish based on our microsatellite allele frequencies were substantial at 42.3 ± 5.2% (s.e.m.) in IKB in 2002–2004, but much smaller at 10.5 ± 4.5% in 2011 (Fig. 3e, Supplementary Table S5). In contrast, the mixing proportion of farmed fish was smaller at 6.1 ± 2.6% in the Uwakai Sea and 3.4 ± 2.2% in Yorishima.

## Discussion

Evidence exists for reduced genetic diversity, survival rates, and reproductive success in hatchery and farmed fish, and for reduced genetic diversity, changes in life history traits and genetic structure in stocked populations. However, this is the first study to demonstrate that the genetic effect of marine stock enhancement diminished with increased size of the wild population. Increases to the seaweed community and reduced fishing efforts were the dominant factors associated with the wild population recovery. Genetic effects of aquaculture were much smaller.

The major spawning ground of KB red sea bream is around natural reefs in the bay-mouth. Fertilized eggs are imported into the KB by tide currents and juveniles settle in the shallow waters of the central and the inner KB (Supplementary Fig. S4a). Seaweed communities in KB should serve as nursery grounds due to abundant copepods^26,27^. In fact, a series of field surveys^28^ found that communities of Hijiki and *Zostera marina* were abundant (Supplementary Fig. S4b,c) with other *Sargassum* species in KB. The results strongly suggest that increase in the wild red sea bream population size were caused by increased seaweed communities on the red sea bream nursery grounds. Our results suggest that rehabilitation of the degraded nursery habitat is indispensable to recover depleted populations. The spawning ground of red sea bream is in the bay mouth and environment degradation is unlikely, suggesting that sound environment of spawning grounds with enough adults is also needed to recover population sizes.

Decreased survival rate of hatchery-reared red sea bream might be caused by the repeated use of broodstock (98 ± 43 fish per year, s. d.) captively reared in the 100-m^3^ concrete tank throughout their lives, where a total of 395 wild and 33 farmed fish were added during 1999–2014 (see Supplementary Information). Under the assumption of a generation time of 4.8 years^29^ (see Supplementary Data), the survival rate of hatchery-reared red sea bream was reduced by 49 ± 6% (s.e.m.) per generation [1 − exp((−0.1391 ± 0.0123) × 4.8) = 0.487 ± 0.058] after 1991 based on the average recapture rate of 1-year and 8-year-old fish. This agreed with the meta-analysis of steelhead, brown trout and Atlantic salmon, which showed captive breeding reduced RRS by ~40% per generation in captivity^11^. However, it is noted that the catch of hatchery-reared red sea bream increased until 1991 before declining (Fig. 1c), showing that fitness of hatchery red sea bream might not be reduced until 3.5 generations (17/4.8 = 3.54) in captive rearing. The threshold number of generations to endure the genetic effect of captive breeding might be higher for red sea bream than salmonids, due to the shorter rearing period in the hatchery (100 days) than salmonids (e.g. one year for steelhead^30^). The long seed production period of steelhead might lead to substantial adaptation in captivity even in a single generation rearing in hatcheries^31^. The exponential decay rate of fitness was higher in the red sea bream than salmonids, suggesting that marine fish can be vulnerable to genetic effects of captive breeding after fitness effects emerged. Exponential decay rates did not significantly differ in 1-year-old and 8-year-old recapture rates. Recaptures of the red sea bream after Age 1 (recruitment to fishery) exponentially decreased until Age 7 and cumulative recapture rates were release-year (cohort) specific and decreased along the years (Supplementary Fig. S5). The result suggests that the rate of fitness reduction in a hatchery-reared population is cohort specific, depending mainly on the duration of captive breeding and the number of broodstock and is constant over time within the cohort, but it exponentially decreases as time duration in captivity increases. Farmed red sea bream mature at age 2 years, and over 11 million (35,398 tonnes (annual farm production)/3 kg (assumed individual weight) = 11.8 million fish) red sea bream spawn in net cages during the spring in the Uwakai Sea. Survival rates of fertilized farmed red sea bream eggs, selected for fast growth since the 1960s (~29 generations assuming 2 year as the generation time), might be much smaller in the wild than hatchery-reared red sea bream born from repeatedly used broodstock reared in the concrete tank since 1974 (~9 generations).

**Figure 4:**
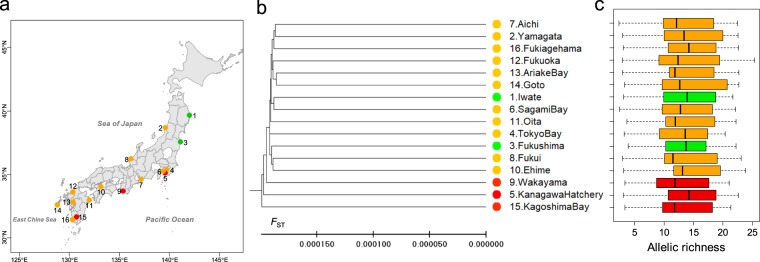
Population structure of red sea bream in Japan. (**a**) Sampling sites, (**b**) EB*F*_ST_ UPGMA dendrogram and (**c**), allelic richness, estimated from 15 microsatellite loci (*n* = 846) by Gonzalez and colleagues^32^. Green points indicate samples from areas where no fish have ever been released, while orange ones show those from areas with hatchery releases. Red points indicate samples from KB, Wakayama with large farm production and a hatchery, for which allele frequencies were different from others^32^.

The high mixing proportion of hatchery fish in IKB indicated substantial gene flow from hatcheries (Fig. 3e). Interestingly, the mixing proportions of hatchery fish in IKB were much higher than *P*_h_ values in IKB (Fig. 1e), implying a trans-generation genetic effect. Recovered mixing proportions showed that the genetic effects diminished across generations. High gene flow from neighboring wild populations might dilute the genetic effect. Our consistent results from microsatellites and mtDNA-CR suggest that females and males equally contributed to reproduction. To examine the robustness of our results, we analyzed published genotype data of 15 microsatellite loci collected from 16 sites over the distribution range in Japan (including a sample from KB; *n* = 846, two individuals with missing genotypes at the *Pma*22 locus were excluded in the analysis)^32^. The Bayesian clustering analysis suggested very weak population differentiation in the Japanese distribution range (*K* = 1, figure not shown). The EB*F*_ST_ population structure was consistent with our result for the genetic effect of aquaculture, which was much smaller than that of hatchery releases, as the sample from Wakayama (where red seabream farms annually produce 1,500 tonnes) was closer to wild populations than to the KB sample (Fig. 4a,b). The dendrogram consistently showed that the genetic effect on allele frequencies remained in KB through at least 2008 (the KB sample was collected by KPFTDC), but allelic richness values were not different in all population pairs (Fig. 4c, *P* = 1.0000, Bonferroni correction). The pairwise EB*F*_ST_ values were very small (0.00039 ± 0.00001, s.d.), showing that effects of hatcheries and aquaculture can be spread farther by very high gene flow. So, more areas would be affected, but none very strongly in a meta-population. High gene flow from wild populations could also facilitate backcrossing to wild populations, which might work to diminish the genetic effects of captive breeding over generations if they were additive^33^. In fact, the genetic effects of the early generation hatchery-reared steelhead on gene expression were additive^34^, and RRS of the wild-born steelhead F_2_ descendants (0.87)^35^ recovered from that of the first-generation hatchery-born fish (0.55)^11^. Salmonids are semelparous with pair-spawning and fecundity is ~3,000 eggs per female. In contrast, red sea bream is iteroparous with multiple episodes of group-spawning during a spawning period, and fecundity is ~1–2 million eggs. The magnitude of diminishing genetic effects of captive propagation could be larger for marine fishes such as red sea bream than salmonids.

**Figure 5:**
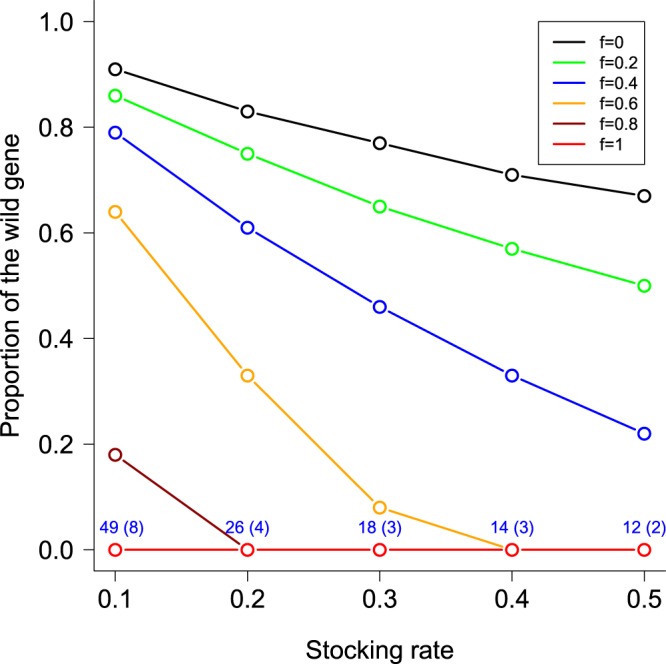
Proportion of the wild gene at equilibrium in a semelparous population under stocking. Drawn from Tables 2, 3 and 4 of Matsuishi and colleagues^38^. *f* shows fitness of hatchery fish. Fitness of wild fish was 1, and that of wild-hatchery hybrids was (1 + *f*)/2. Numbers in the graph show the number of generations at which more than 99% (50%) of the wild genes in the population are expected to be replaced by the hatchery gene.

The genetic effect in a population depends on the number of parents that are captively bred. If only a few parents are used, the Ryman-Laikre (R-L) effect^36^ could reduce the effective population size, and hence reduce genetic diversity, in a combined captive–wild system. Typically, not all parents contribute to production of fertilized eggs in spawning tanks and farm net-cages. In such cases, the R-L effect can be more substantial for even modest stocking rates^37^. Our results suggest that the R-L effect occurs even for hatchery fish with reduced fitness. The genetic effect can be more persistent for cases with equal and/or similar fitness of artificially-produced animals compared to wild ones. Theoretical work of Matsuishi and colleagues^38^ predicted the rate of replacement of the wild gene by the hatchery gene at a locus in a closed population at equilibrium. Their model assumed that wild and hatchery fish are semelparous with equal sex ratio and have the genotypes *WW* and *HH*, respectively, at a locus. We summarized the results of Matsuishi *et al*. for cases of the additive effect (Fig. 5), which showed that the hatchery gene spread within several generations depending on fitness of hatchery-reared animals (*f*) and stocking rates (*R*). Even if *f* = 0, portions of the wild gene will be replaced by the hatchery gene by the hybrids. Complete replacement (=the wild gene <1%) can occurs for cases of *f* ≥ 0.6 and *R* ≥ 0.4. Assuming *R* = 0.1 and equal fitness for wild and hatchery fish (*f* = 1), more than 99% (50%) of wild genes are expected to be replaced by hatchery genes in 49 (8) generations^38^ (Fig. 5). Reduced fitness of farmed Atlantic salmon^13,14^ and altered age and size at maturation in Atlantic salmon populations^18^ suggested that intended selection for fast growth and other traits simultaneously might select genes related to fitness. These results imply that fitness effects on Atlantic salmon populations may arise in the near future if escapement from fish farms continues. Reduced fitness of the red sea bream shows that even unintended selection in captive breeding can select genes related to fitness. The fitness effect on KB population is masked in large wild populations.

## Conclusions

The biological function of hatcheries is to compensate for natural reproduction by artificial propagation techniques. Our results indicate that hatcheries for enhancement and conservation of populations cannot be successful over the long term unless sufficient effort to reduce fishing efforts and to rehabilitate the degraded nursery and spawning habitat is also made.

## Methods

### Causal factors of recovery of wild population

We analyzed the causes of increased catches of wild red seabream in KB for the recovering period (1989–2015), where the objective variable was wild red seabream catch, and the explanatory variables were harvests of Hijiki (edible brown algae), SST (sea surface temperature), fishing effort (total number of gill nets, pole and lines, longlines, and set nets operated and targeting red seabream in the bay), *P*_h_ (proportion of hatchery fish in the landings), and accumulated reclaimed land. We estimated fishing efforts on red sea bream (1989–2015) using a simple regression based on total fishing efforts in KB by five years (1973–2013 Fishery Census, Supplementary Data). Wheareas the catch in a given year was created by the fishing effort of that year, fishing effort could affect reproduction in the previous several years, and this effect was included in the catch. Rational determination of time-lags for the reproduction effect was difficult because of overlapping generations in the catch. Therefore, we used fishing effort data without considering time-lags. The regression analyses used R function lm, and step(lm) for stepwise selection of the explanatory variables by AIC.

### Fish samples

Eight samples totalling 642 red sea bream from six geographical regions were collected between 2002 and 2011 (497 wild, 45 hatchery, and 100 farmed fish), of which 167 fish collected during 2002 and 2004 were previously published samples^39^ The 2002 and 2004 samples were obtained from several fishing ports in the East China Sea (ECS0203) where few releases had been made, and the IKB (IKB0204), including wild fish without DIE. Hatchery-released individuals recaptured in the bay (HA0204) were identified DIE, and wild fish without DIE. In addition to the previously published samples, we collected new wild fish samples from the same IKB (IKB11) and East China Sea (ECS10) sites to compare the genetic effects in KB in 2009–2011 to those in 2002–2004. Hatchery fish were not collected in 2011 because catch was very low, but hatchery fish were produced from the same brood stock during this period (see Supplementary Information). Therefore, IKB04 and IKB11 were affected by the repeatedly-used hatchery broodstock, which can be represented by HA0204. We also collected wild fish samples from Yorishima (YR10), Okayama Prefecture, Uwakai Sea (UK09), Ehime Prefecture, and a sample from a fish farm in Uwakai Sea (FA09) to evaluate the genetic effects of escapees and spawning of farmed fish. Selective breeding of red sea bream for fast growth has been carried out since the 1960s, leading to successful domestication of a major strain (see Supplementary Information), which is widely used in western Japan, including Uwakai Sea where UK09 was collected. All fish samples bought from fishers and a fish farm were sent with ice to our laboratory at Tokyo University of Marine Science and Technology. Muscle tissue from each specimen was stored in 99.5% ethanol for DNA extraction.

### DNA extraction, sequencing and genotyping

Genomic DNA was extracted using the QuickGene Mini-80 (Wako Pure Chemical, Osaka Japan), according to the manufacturer’s instructions. DNA fragments corresponding to the CR [~500 base pairs (bp)] were amplified by polymerase chain reaction (PCR) according to our previous study^39^. The 465-bp mtDNA control regions (mtDNA-CR) were sequenced in 313 fish and aligned with our previously published sequences of 180 fish^40^. The sequences were aligned using ClustalX^41^. Haplotypes were defined based on the sequence data using DnaSP ver. 5.10^42^.

We genotyped 642 fish and examined nine microsatellite markers developed for *Pagrus major*^43^ (*Pma*1, *Pma* 2, *Pma* 3, *Pma* 4, and *Pma* 5) and *Pagrus auratus*^44^ (*GT*2, *GT*4, *GA*2*A*, and *GA*2*B*), which is closely related to *P. major*. An excess number of alleles per locus and a wide range in base pair length were detected at the *Pma*4 locus; thus, identifying some alleles, particularly long alleles, was rather difficult^43^. We could not amplify the DNA fragments or detect the primer dimer in the *GT*2, *GT*4, and *GA*2*B* loci. Thus, four loci (*Pma*4, *GT*2, *GT*4, and *GA*2*B*) were excluded. All loci were scored for all individuals, including previously published samples by capillary electrophoresis using an ABI PRISM 3130xl Genetic Analyzer with fluorescent dye-labeled primers. The thermal cycles for PCR amplification were conducted in 10 µL aliquots of a mixture containing 1.0 µL genomic DNA as template (~0.1 µg), 0.1 µL KOD FX, 0.1 µL of 10 µM primers, 5 µL 10 × Ex PCR buffer (8 mM MgCl_2_), 2.0 µL dNTP mixture, and 1.7 µL sterile water using a thermal cycle profile published previously^43,44^. Genotype data quality was evaluated using Microchecker^45^ to detect scoring errors and null alleles. Linkage disequilibrium and conformation to Hardy–Weinberg equilibrium (HWE) were tested using GENEPOP 4.2^46^ (linkage disequilibrium: 5,000 dememorizations, and 100 batches, 5,000 iterations per batch; HWE: 10,000 dememorizations, 100 batches, and 10,000 iterations per batch). A total of 157 alleles were found at the five microsatellite loci. Scoring errors caused by stuttering, large allele dropout, or null alleles were not detected. Linkage equilibrium was observed for all pairs of loci in all wild samples and HA0204, but linkage disequilibrium was found in UK09 (between *Pma*1 and *Pma*2) and in FA (for seven of 10 locus pairs). HWE was observed for all loci in IKB11. Departures from HWE were observed in three loci in the FA09 and HA0204 samples, and in one locus in all wild fish samples except IKB11, but HWE was confirmed in all loci and samples after Bonferroni correction (Supplementary Fig. S6).

### Population genetics analysis

The number of haplotypes, haplotype diversity^47^, and nucleotide diversity^48^ were estimated using DnaSP. Haplotype richness was calculated using CONTRIB^49^, with the smallest sample size for rarefaction. Allelic richness and heterozygosity were calculated for each locus and population using FSTAT 2.9.3.2^50^ and Arlequin 3.5.1.3^51^, respectively. Samples were rarefied to the smallest sample size to determine allelic richness. The exact test for population differentiation based on the haplotype and allele frequencies was performed using the Markov chain procedure (5,000 dememorization, 100 batches, 5,000 iterations per batch), as implemented in Genepop. We estimated genetic differentiation between populations in terms of Wright’s *F*_ST_^52^. We estimated the posterior means of pairwise *F*_ST_ values (EB*F*_ST_)^53^ for high gene flow species^54^ between all population pairs based on the haplotype and allele frequencies using EBFST function in R package FinePop1.4.1. Based on the EB*F*_ST_ values, we drew UPGMA trees using MEGA7^55^. We also ran STRUCTURE^56^ under a burn-in of 100,000 iterations, followed by 500,000 Monte Carlo–Monte Carlo (MCMC) repetitions for the number of putative original populations *K* = 1–5. We used the conditional-likelihood method for genetic stock identification^57^ using BASEMIX^58,59^ and ONCOR^60^. Standard errors of the mixing proportion estimates were calculated considering the variances in the gene frequencies of the baseline populations using BASEMIX. The 95% confidence intervals for the mixing proportion estimates were calculated with 1,000 bootstraps in ONCOR. Both programs gave similar estimates for the moderate mixing proportion values (Supplementary Table S5). ONCOR provided much smaller estimates for our cases of <10%, which resulted in larger estimates for the major baseline population. BASEMIX calculates standard errors of the mixing proportion estimates by explicitly considering the variances in the gene frequencies of the baseline populations. We used estimates of the mixing proportions obtained from BASEMIX for subsequent analyses. We estimated the mixing proportions of hatchery fish (HA0204) in the IKB0204 and IKB11 and of farmed fish (FA09) in the UK09 and YR10.

## Supplementary information

Supplementary Information

Supplementary Data

## Data Availability

The datasets generated during and/or analysed during the current study are available in Supplementary Data. The haplotype sequences of the red seabream are deposited in GenBank under accession numbers AB909131–AB909368, and the red seabream microsatellite genotypes are available from the Dryad Digital Repository (datadryad.org; 10.5061/dryad.4pg1vv4).
