# Supplementary material for: Rigorous monitoring of a large-scale marine stock enhancement program demonstrates the need for comprehensive management of fisheries and nursery habitat
Source: Sci Rep. 2019 Mar 27;9:5290. doi: 10.1038/s41598-019-39050-3 (PMC6437203; doi:10.1038/s41598-019-39050-3)
Supplement: Supplementary file 1 — Supplementary Information [file 41598_2019_39050_MOESM1_ESM.pdf]

Supplementary Information for:

Rigorous monitoring of a large-scale marine stock enhancement program demonstrates the need for comprehensive management of fisheries and nursery habitat

Shuichi Kitada[1*], Kaori Nakajima[1], Katsuyuki Hamasaki[1], Hirotoshi Shishidou[2], Robin S. Waples[3], and Hirohisa Kishino[4]

[1]Tokyo University of Marine Science and Technology, Tokyo 108-8477, Japan. [2]Kagoshima Prefectural Fisheries Technology and Development Center, Kagoshima 891-0315, Japan. [3]Northwest Fisheries Science Center, NOAA, Seattle, WA 98112, USA. [4]Graduate School of Agriculture and Life Sciences, The University of Tokyo, Tokyo 113-8657, Japan.

*Shuichi Kitada, E-mail: kitada@kaiyodai.ac.jp

This PDF file include three Supplementary Information:

A. Red sea bream stock enhancement in Kagoshima Bay and its aquaculture in Japan
B. Supplementary Tables S1–S5
C. Supplementary Figures S1–S6

A. Red sea bream stock enhancement in Kagoshima Bay and its aquaculture in Japan

The stock enhancement program in Kagoshima Bay (KB). KB is a semi-closed bay of 1,129 km^2 with an 8.7 km wide mouth, located near southern Kyushu. Total coastline is 330 km, and ~60% has been modified artificially. The active volcano Sakura-jima divides the bay into inner and central bays across the Nishi-Sakurajima Channel with a width of 1.9 km and depth of 40 m. The inner bay is a gigantic 250-km^2 volcanic caldera, with mean depth of 140 m, and is shaped like a deep pond with a maximum depth of 206 m[A1]. The central bay is 580 km^2 with mean depth of 126 m and maximum depth of 237 m. A total of 27.33 million hatchery-reared red sea bream born from captively-reared broodstock were released into 18 sites in the semi-closed KB from 1974 to 2016 with the goal of increasing depleted fishery production. About 130 non-local red sea bream have been maintained in a 100-m^3 concrete tank for natural spawning of broodstock at the Kagoshima Prefectural Hatchery. Progeny of the founder parents are repeatedly used as broodstock. A total of 395 wild (1999–2014), 33 farmed (2011–2012), and 2-year-old hatchery fish produced from broodstock were added to the broodstock in 2009. The number of parental fish used for seed production varies between 45 and 187 every year (Fig. A1).

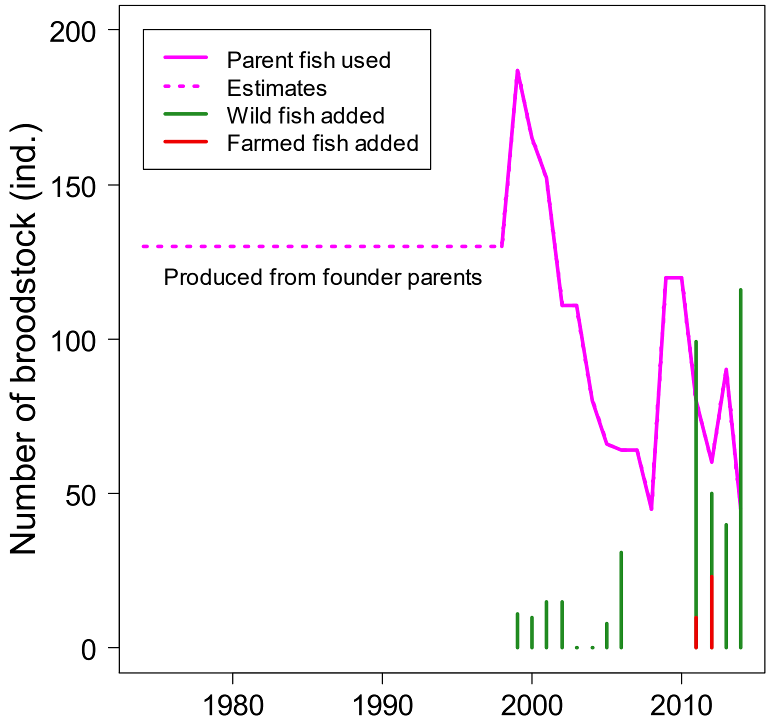

Fig. A1. Broodstock used for seed production at Kagoshima Prefectural Hatchery.

Natural spawning occurs in a 100-m^3 concrete tank and fertilized eggs are collected for seed production (Fig. A2a, b). Hatched larvae are reared for about 100 days: 50 days in 100-m^3 concrete tanks (Fig. A2c) until they reach total length (TL) of 25–30 mm and then for an additional 50 days in floating net sea cages until their release at about 70 mm TL. Rearing in net cages was discontinued in 2000, and larvae are now reared for 90 days in concrete tanks until release at 60 mm TL. Before release, 110–1,200 fish were sampled, and their nostrils were checked to estimate the proportion with the deformity of the internostril epidermis (DIE) in the sample, which is used to identify hatchery fish at landing. DIE is a morphological peculiarity of hatchery fish caused by procedural conditions in hatcheries[A2] and is not found in wild fish[A3]. The incidence of DIE was very high with large variations (77.0 ± 18.1%) during 1989–2015. The number of seeds released exceeded 1 million in 1981 and reached the historical maximum of 1.297 million in 1987. Releases remained at ~1 million until 1992, but then decreased to ~460 thousand in 2015, mainly due to a budget cut.

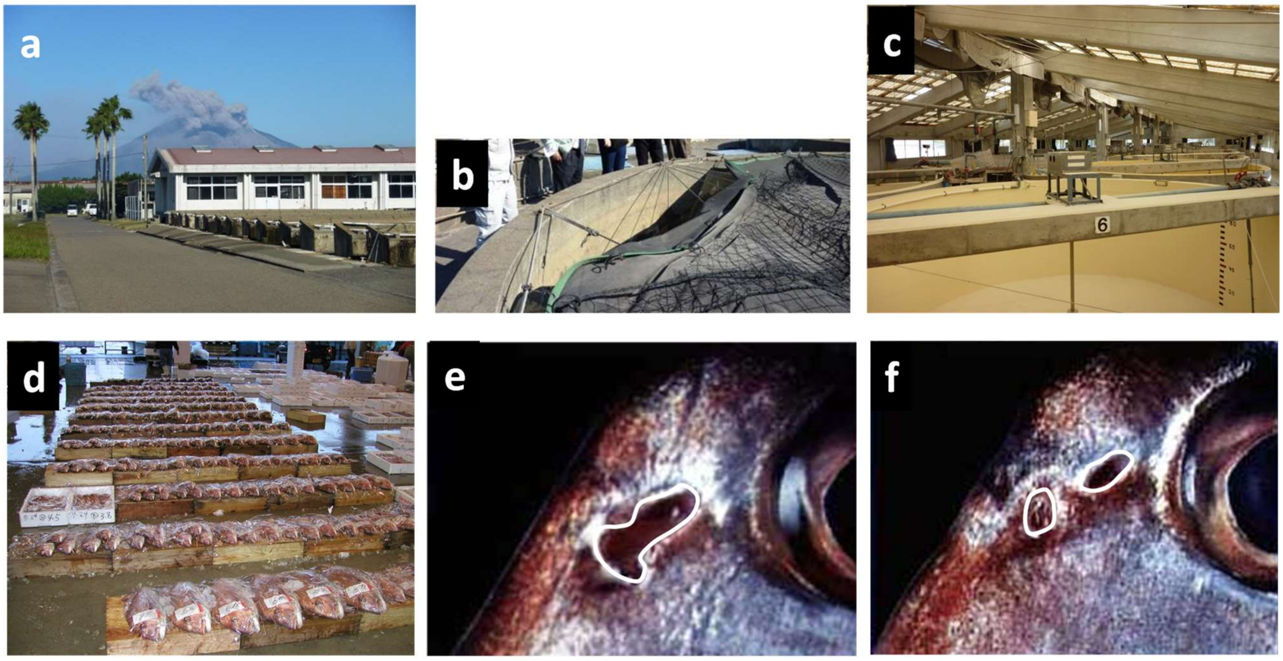

Fig. A2. Photographs from red sea bream enhancement at Kagoshima Bay. **a**, Hatchery. **b**, 100 ton broodstock rearing tank, and **c**, seed production tanks. **d**, Commercial landings at the Kagoshima City Fish Market. **e**, Hatchery-reared with the deformity of the internostril epidermis and **f**, wild red sea bream identified at market. Nostrils were indicated by white lines.

Fish market survey and fishing effort. Red sea bream are caught by pole and line, gochi-nets (surrounding seine), gill nets, and long lines in KB. More than 80% of red sea bream caught in KB

are landed at the Kagoshima City Fish Market (KCFM). Commercial landings of wild and hatchery fish have been intensively monitored at fish markets by Kagoshima Prefectural Fisheries Technology and Development Center (KPFTDC). Fish are displayed according to body size (Fig. A2d). KPFTDC staff surveyed all fish landed at KCFM 1–3 days/week since 1989[A4]. The body weights of all fish landed on survey days were measured, and the name of the fisherman who caught the fish was recorded. To evaluate the effectiveness of release, 1,598,560 thousand red sea bream landed on fish markets have been checked for DIE after 1989 mainly at KCFM. Hatchery-released fish recaptured in KB were identified by DIE (Fig. A2e, f), and all fish were checked for DIE. Red sea bream caught by fishermen on the Osumi Peninsula were not usually landed at the KCFM. The same KCFM survey has been conducted at several fish markets in this area to estimate total landings of hatchery and wild fish. In addition, the same survey was conducted at four to eight fish markets outside KB to estimate stocking effectiveness. Age composition and mean body weights of hatchery and wild fish by age were estimated based on an age–weight key, and the total numbers of landings were estimated for hatchery and wild fish based on commercial landings[A4].

The total number of fishers including fishery enterprise (hereafter, fishers) and red sea bream fishing efforts (=total number gill nets, pole and lines, long lines and set nets operated targeting for red sea bream) in KB has decreased consistently since 1983 (Fig. A3).

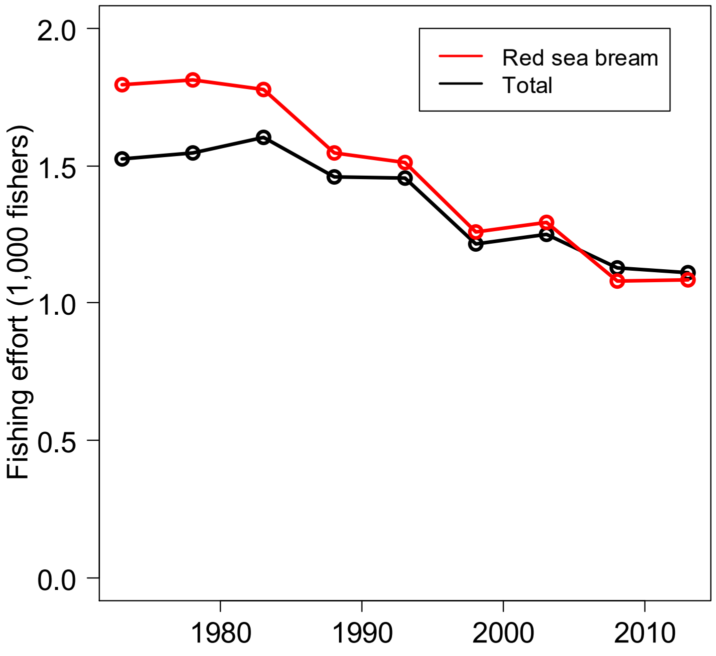

Fig. A3. Total number of fishers and fishing effort targeting red sea bream in KB (1983-2013, every five years from Japan Fishery Census[A5]).

Bias of DIE in evaluating effects of releases. DIE rates have varied over the years, and no correlation was found with recapture rates ($r = 0.31$, $P = 0.1319$) (Fig. A4), suggesting that DIE might not be caused by the genetic effects of captive breeding of parental fish and does not affect survival rate after release. The mean incidence of DIE was very high between 1989 and 2013 (79.0 ± 16.7), but we could not exclude the possibility that hatchery fish with normal nostrils were included in the samples. Therefore, the stocking effectiveness and the genetic effects could have been overestimated. Here, we examined the bias for the genetic effects in IKB0204. IKB0204 individuals (1–3-year-old fish) hatched in 1999–2003. The rate of DIE was 81.5 ± 22.5% for fish spawned during these years. The mean proportion of hatchery fish in IKB landings was 11.7 ± 1.10% in 2002–2004 when the IKB0204 sample was collected, which was estimated based on the DIE rates of fish. The sample could include hatchery fish with normal nostrils. If so, the probability of hatchery fish with normal nostrils in the random sample taken from the landings was (1 − 0.815) × 0.117 = 0.0216. In the same way, IKB11 individuals (1–3-year-old fish) hatched in 2008–2010 had a DIE rate of 61.0 ± 15.3%. The mean proportion of hatchery fish in the IKB landings was 1.9% in 2011, and the probability of hatchery fish with normal nostrils in the random sample taken from the landings was (1 − 0.61) × 0.019 = 0.0074. The probability of hatchery fish with normal nostrils included in the IKB0204 and IKB11 samples was thus less than ~2%, which should not substantially affect the results of this study.

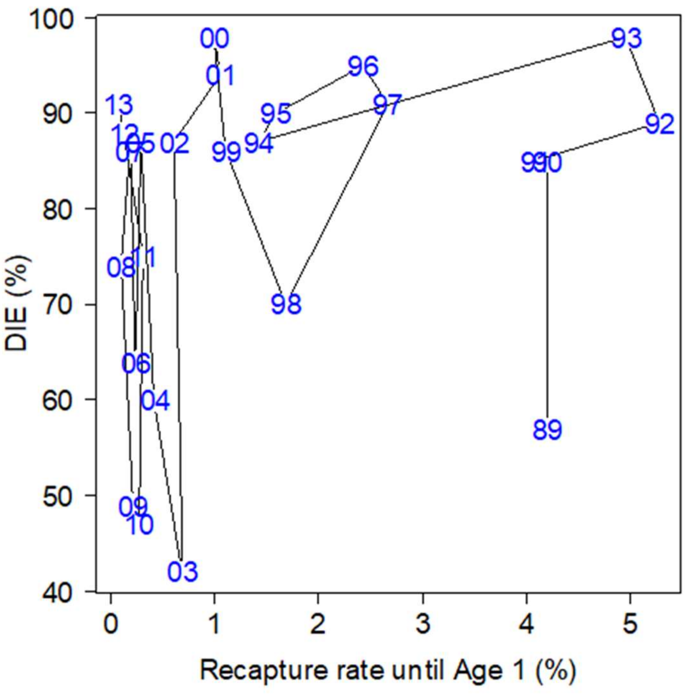

Fig. A4. Correlation between recapture rates and rates of the deformity of the inter-nostril epidermis (DIE) in Kagoshima Bay. The numbers in the graph are years (1989–2013).

Environment data. We organized the land reclamation data from 1965 to 2015, as reported to the Kagoshima Prefectural Government, and collected catch statistics for edible brown alga (Hijiki, *Sargassum fusiforme*) from 1975 to 2015[A6]. We estimated Hijiki community sizes in 2006 and 2013 by multiplying community size observed in 1976 and 1978 by the ratio of the 1978 catch to the 2006 catch and the ratio of the 2006 catch to the 2013 catch using the *Zostera*, *Sargassum*, and Hijiki community sizes surveyed in KB during 1976 and 1978[A7] and the community sizes of *Zostera* and *Sargassum* surveyed in 2006 (KPFTDC, unpublished data). In the calculation, we assumed that harvest pressure on Hijiki was constant during 1976–2006 when the harvest was low, but that it increased 1.51 times during 2006–2012, which was estimated from the number of fishers targeting KB seaweeds (KPFTDC). We organized annual mean SST values in KB (5 m depth) observed by KPFTDC from 1980 to 2016 (latitude 31.50 N and longitude 130.58 E).

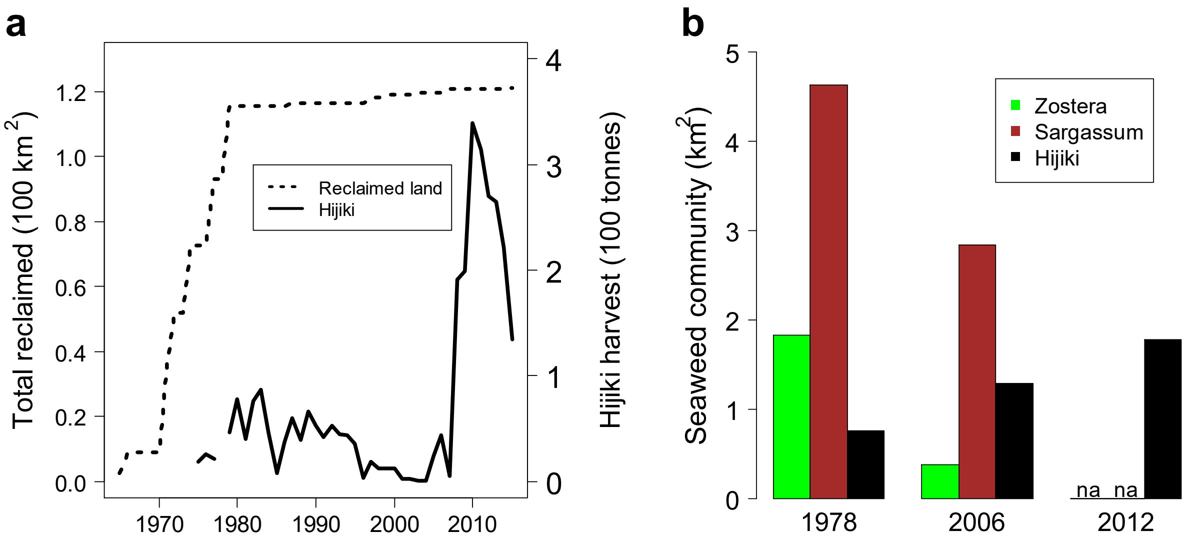

Fig. A5. Environmental changes in Kagoshima Bay (KB). **a**, Cumulative reclaimed land and harvest of Hijiki (edible brown alga, *Sargassum fusiforme*) in KB (1965-2015). **b**, Changes in KB community sizes of *Zostera*, *Sargassum*, and Hijiki. na, data not available.

Substantial land reclamation was carried out in KB in the 1970s, but almost no reclamation was conducted during 2008 and 2015 (Fig. A5a). Catch during 2007–2012 could include a negligible amount of Wakame (*Undaria pinnatifida*), but we treated this as Hijiki harvest in our analysis. Landings of Hijiki have increased remarkably since 2008. The *Zostera* and *Sargassum* communities were abundant in KB in the 1970s but decreased in 2006. In contrast, the Hijiki community was relatively small in the 1970s but has increased since 2006 (Fig. A5b).

Sea surface temperature (SST) in the north part of the East China Sea, which includes KB, showed an increasing trend of +1.21 ± 0.25°C for the past 100-year average (Fig. A6a). The annual average SST is also increasing in KB, with a mean of 21.03°C for 1980–1984 and 21.53°C for 2012–2016 (Fig. A6b). Global warming effects on seaweed beds[A8] and a rapid shift from temperate to tropical *Sargassum* species in western Japan[A9] suggest that tropical *Sargassum* species have increased in number in KB. In fact, landings of Hijiki have increased remarkably since 2008, and their communities have increased in size recently (Fig. A5b). A shift from dominant warm-temperate *Sargassum* species to a mixture of warm-temperate and subtropical species has also been observed in KB[A10].

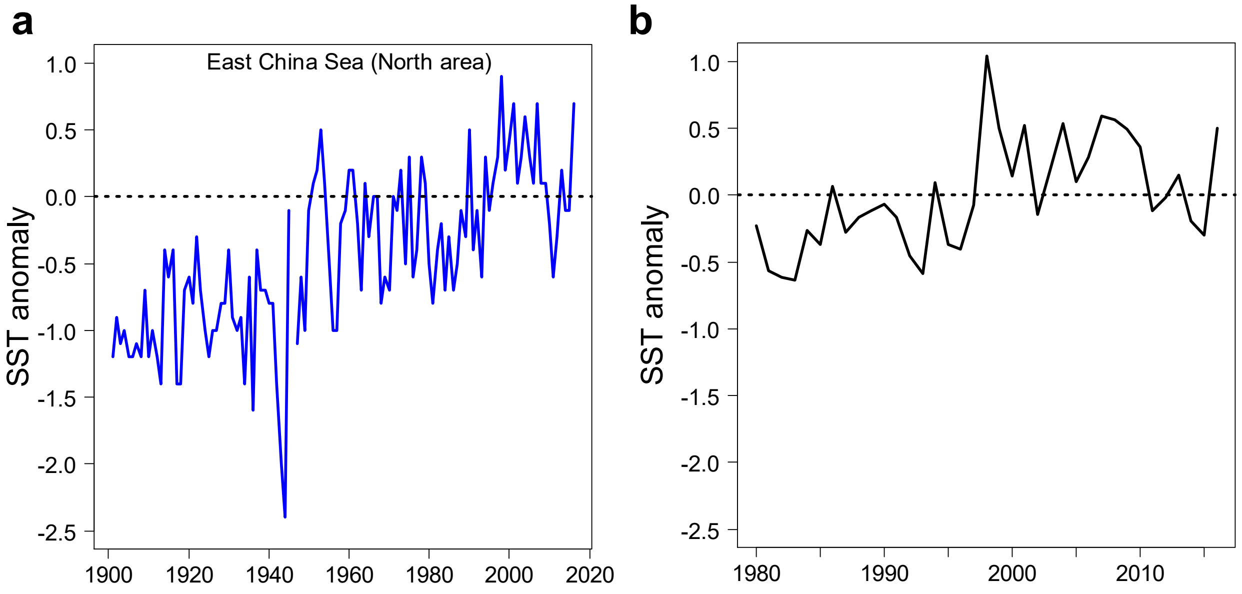

Fig. A6. Changes in annual mean sea surface temperature (SST anomaly) in **a**, the north area of the East China Sea (data, Japan Meteorological Agency, http://www.data.jma.go.jp/kaiyou/data/shindan/a_1/japan_warm/japan_warm.html, 1900–2016) and **b**, in Kagoshima Bay (1980–2016).

Red sea bream aquaculture in Japan.

Red sea bream has the highest production of aquaculture fish in Japan that uses artificially-produced seed. The Uwakai Sea (UK) has a sawtooth coastline and faces Bungo Channel. Annual red sea bream cage culture production is the highest in Japan in this area owing to the geographical features (Fig. A7). Aquaculture production in 2014 was 35,398 tons (57.4% of all of Japan), whereas red sea bream catch from capture fisheries was only 1,217 tons in Ehime Prefecture[A6]. Farm production was 29 times greater than that from wild catch. The net cages generally used in UK was closed and submerged except during feeding times to avoid damage by storms (Fig. A8). Fish farmers used artificially produced seeds from seed production companies. Selective breeding for fast growth has been carried out since the 1960s and successfully domesticated a major strain[A11]. The strain is widely used in western Japan, which requires only 1.5 years to rear to market size (~1.5 kg per individual), whereas 3 years are required for wild fish[A12]. These fish mature at age 2 years and spawn in Uwakai Sea net cages during spring. In contrast, no aquaculture or stocking are conducted in Okayama Prefecture, including Yorishima (YR).

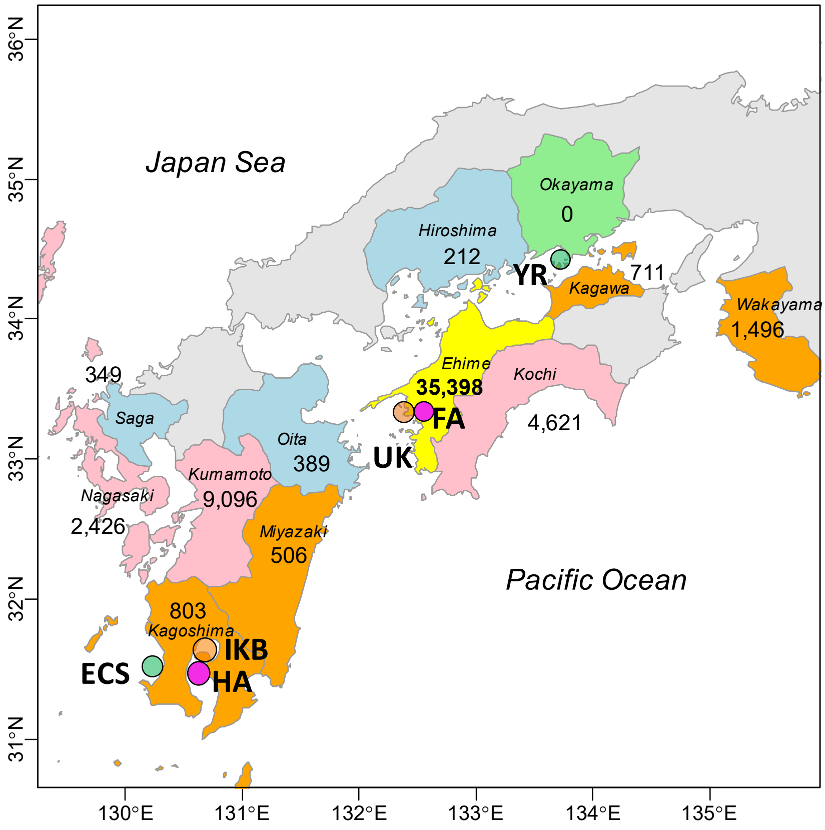

Fig. A7. Red sea bream aquaculture production (tons) by prefecture in 2014[A6] with sampling sites for genetic monitoring. The five colours show the production scale in each prefecture. Yellow, >35,000 tonnes. Pink, >4,500 tonnes. Orange, >500 tonnes. Blue, <500 tonnes. Green, no aquaculture production.

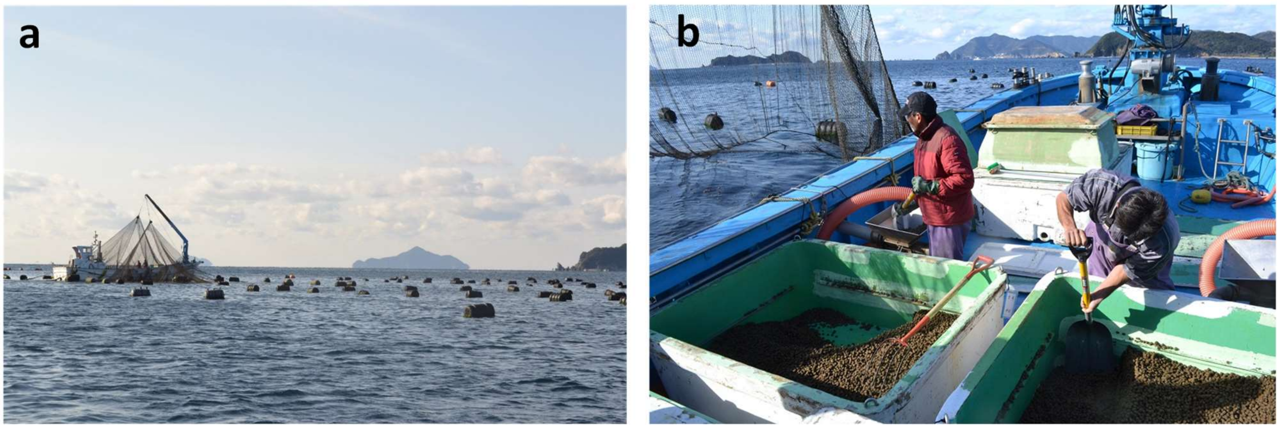

Fig. A8. Photographs of red sea bream aquaculture in the Uwakai Sea (UK). **a**, A net cage, closed and submerged except during feeding times to avoid damage by storms. **b**, Feeding.

Supplementary References

A1. Shiihara, H. Stocking effectiveness and perspectives in Kagoshima Bay. In *Sea Farming Technology of Red Sea Bream* (eds. Tanaka, M., Matsumiya, Y.) pp. 106–126 (Koseisha Koseikaku, 1986). [in Japanese]

A2. Mana, R. R. & Kawamura G. A comparative study on morphological differences in the olfactory system of red sea bream (*Pagrus major*) and black sea bream (*Acanthopagrus schlegeli*) from wild and cultured stocks. *Aquaculture* **209**, 285–306 (2002).

A3. Sobajima, N., Munekiyo, M. & Funata, H. Possibility of differentiation between the artificially-released and the wild red sea bream by means of the lack of the internostril epidermis. *Bul. Kyoto Inst. Ocean. Fish. Sci.* **10**, 35–40 (1986). [in Japanese with English abstract]

A4. Shishidou, H. & S. Kitada. Stocking effectiveness of red sea bream *Pagrus major* in Kagoshima Bay, Japan. *Nippon Suisan Gakkaishi* **73**: 270–277 (2007). [in Japanese with English abstract]

A5. Ministry of Agriculture, Forestry and Fisheries. *The Census of Fisheries 1973–2013* (Ministry of Agriculture, Forestry and Fisheries, 2015). [in Japanese]

A6. Ministry of Agriculture, Forestry and Fisheries. *Annual Statistics of Fisheries and Aquaculture Production in 1975–2015* (Association of Agriculture and Forestry Statistics, Tokyo) (1977–2017). [in Japanese]

A7. Setoguchi, Y., et al. *Distributions of Seaweeds Communities and Tidelands in Kagoshima Prefecture Coasts* (*Seikai Nat. Fish. Inst.*, 1981). [in Japanese]

A8. Komatsu, T. et al. Possible change in distribution of seaweed, *Sargassum horneri*, in northeast Asia under A2 scenario of global warming and consequent effect on some fish. *Mar. Poll. Bull.* **85**, 317–324 (2014).

A9. Yamasaki, M. et al. Drifting algae and fish: Implications of tropical Sargassum invasion due to ocean warming in western Japan. *Estuar. Coast. Shelf Sci.* **147**, 32–41 (2014).

A10. Tanaka, T. et al. Distribution and characteristics of seaweed/seagrass community in Kagoshima Bay, Kagoshima Prefecture, Japan. *Nippon Suisan Gakkaishi* **79**: 20–30 (2013). [in Japanese with English abstract].

A11. Murata O, et al. Selective breeding for growth in red sea bream. *Fish. Sci.* 62, 845–849 (1996).

A12. Sawayama, E. & Takagi, M. Genetic diversity and structure of domesticated strains of red sea bream, Pagrus major, inferred from microsatellite DNA markers. *Aquacult. Res*, doi: 0.1111/are.12498 (2014).

B. Supplementary Tables S1–S5

Supplementary Table S1. Samples for genetic monitoring of the Kagoshima Bay red sea bream st

Location (East to West, in Fig. 3)	Abbrevi-ation	Body length (cm)	Age	Sampling date	San
					microsatell
Yorishima	YR10	10.2±0.6	0	Sep., 2010	98
Uwakai Sea (farm)	FA09	18.7±1.5	1	June, 2009	100
Uwakai Sea	UK09	20.2±2.3	1–2	July–Aug., 2009	116
Kagoshima Bay	HA0204	24.7±5.7	1–3	Apr., 2002–June, 2004	45 [a)]
Inner Kagoshima Bay	IKB0204	25.4±6.0	1–3	Apr., 2002–June, 2004	64 [a)]
Inner Kagoshima Bay	IKB11	26.1±2.0	1–3	Mar., 2011	61
East China Sea	ECS0203	25.8±3.8	1–3	Sep., 2002–Dec., 2003	58 [a)]
East China Sea	ECS10	26.6±1.2	1–3	Dec., 2010	100
Total					642

All samples were newly genotyped and sequenced including tissue samples from **a**, Shishidou et al. (20
Hamasaki et al. (2010)[40] were used.

Supplementary Table S2. Red sea bream genetic diversity indices

Sample	Farmed/ Hatchery /Wild	mitochondrial DNA (D-loop)				microsatellite loci		
		n	h_r	h ± s.e.	π ± s.e.m.	n	Ar ± s.d.	H_0 ± s.d.
YR10	W	59	33.01	0.996 ± 0.004	0.0242 ± 0.0012	98	20.78 ± 3.79	0.851 ± 0.120
FA09	F	94	3.71	0.622 ± 0.039	0.0145 ± 0.0008	100	8.35 ± 2.52	0.676 ± 0.104
UK09	W	104	33.08	0.997 ± 0.002	0.0256 ± 0.0007	116	19.39 ± 4.32	0.838 ± 0.136
HA0204	H	36	7.00	0.851 ± 0.029	0.0155 ± 0.0010	45	14.00 ± 3.77	0.800 ± 0.114
IKB0204	W	58	21.76	0.960 ± 0.012	0.0232 ± 0.0008	64	18.69 ± 3.98	0.822 ± 0.095
IKB11	W	56	30.02	0.988 ± 0.007	0.0253 ± 0.0008	61	20.51 ± 3.86	0.892 ± 0.058
ECS0203	W	86	32.80	0.997 ± 0.002	0.0252 ± 0.0007	58	21.11 ± 4.97	0.865 ± 0.088
ECS10	W	-	-	-	-	100	21.72 ± 4.79	0.874 ± 0.097

n, sample size; hr, haplotype richness; *h*, haplotype diversity; π, nucleotide diversity; *Ar*, allelic richness; H_0, observed heterozygosity; s.e.m., standard error; s.d., standard deviation.

Supplementary Table S3. Red sea bream mitochondrial DNA control region population differentiation. *P*-values for the population differentiation test (upper diagonal) and empirical Bayes pairwise F_{ST} estimates (lower diagonal)

	YR10	FA09	UK09	HA0204	IKB0204	IKB11	ECS0203
YR10		**0.0000**	0.0640	**0.0000**	**0.0000**	**0.0023**	0.2169
FA09	0.0070		**0.0000**	**0.0000**	**0.0000**	**0.0000**	**0.0000**
UK09	0.0018	0.0078		**0.0000**	**0.0001**	0.1007	0.7383
HA0204	0.0024	0.0048	0.0028		0.0135	**0.0000**	**0.0000**
IKB0204	0.0020	0.0055	0.0023	0.0019		**0.0023**	**0.0000**
IKB11	0.0018	0.0071	0.0018	0.0023	0.0019		0.1985
ECS0203	0.0018	0.0076	0.0017	0.0028	0.0022	0.0018	

Bold type indicates a significant difference at $P < 0.05$ after Bonferroni correction.

Supplementary Table S4. Red sea bream microsatellite differentiation. *P*-values for the population differentiation test (upper diagonal) and empirical Bayes pairwise F_{ST} estimates (lower diagonal)

	YR10	FA09	UK09	HA0204	IKB0204	IKB11	ECS0203	ECS10
YR10		**0.0000**	0.3384	**0.0000**	**0.0004**	0.0150	0.3366	0.1225
FA09	0.0339		**0.0000**	**0.0000**	**0.0000**	**0.0000**	**0.0000**	**0.0000**
UK09	0.0031	0.0311		**0.0000**	**0.0000**	0.1089	0.0269	0.0104
HA0204	0.0072	0.0297	0.0070		**0.0000**	**0.0000**	**0.0000**	**0.0000**
IKB0204	0.0043	0.0286	0.0045	0.0054		**0.0001**	**0.0000**	**0.0005**
IKB11	0.0041	0.0288	0.0040	0.0076	0.0047		0.0454	0.1641
ECS0203	0.0038	0.0295	0.0039	0.0076	0.0048	0.0042		0.0461
ECS10	0.0034	0.0318	0.0037	0.0066	0.0042	0.0038	0.0037	

Bold type indicates a significant difference at $P < 0.05$ after Bonferroni correction.

Supplementary Table S5. Mixing proportion of hatchery fish (HA0204) in the inner Kagoshima Bay (IKB0204 and IKB11) and farmed fish (FA09) in Yorishima (YR10) and the Uwakai Sea (UK09)

Mixed population (sample size)	Baseline populations (sample size)	BASEMIX		ONCOR	
		Estimate	s.e.m.	Estimate	95%CI
IKB0204 (64)	HA0204 (45)	0.4227	0.0520	0.4506	0.279–0.619
	ECS0203 (58)	0.5476	0.0520	0.5494	0.381–0.721
IKB11(61)	HA0204 (45)	0.1052	0.0449	0.0010	0.000–0.089
	ECS10 (100)	0.8768	0.0449	0.9990	0.911–1.000
YR10 (98)	FA09 (100)	0.0342	0.0222	0.0039	0.000–0.029
	ECS10 (100)	0.9403	0.0222	0.9961	0.971–1.000
UK09 (116)	FA09 (100)	0.0614	0.0219	0.0257	0.000–0.058
	ECS10 (100)	0.9231	0.0219	0.9743	0.942–1.000

s.e.m., standard error; CI, confidence interval.

C. Supplementary Figures S1–S6

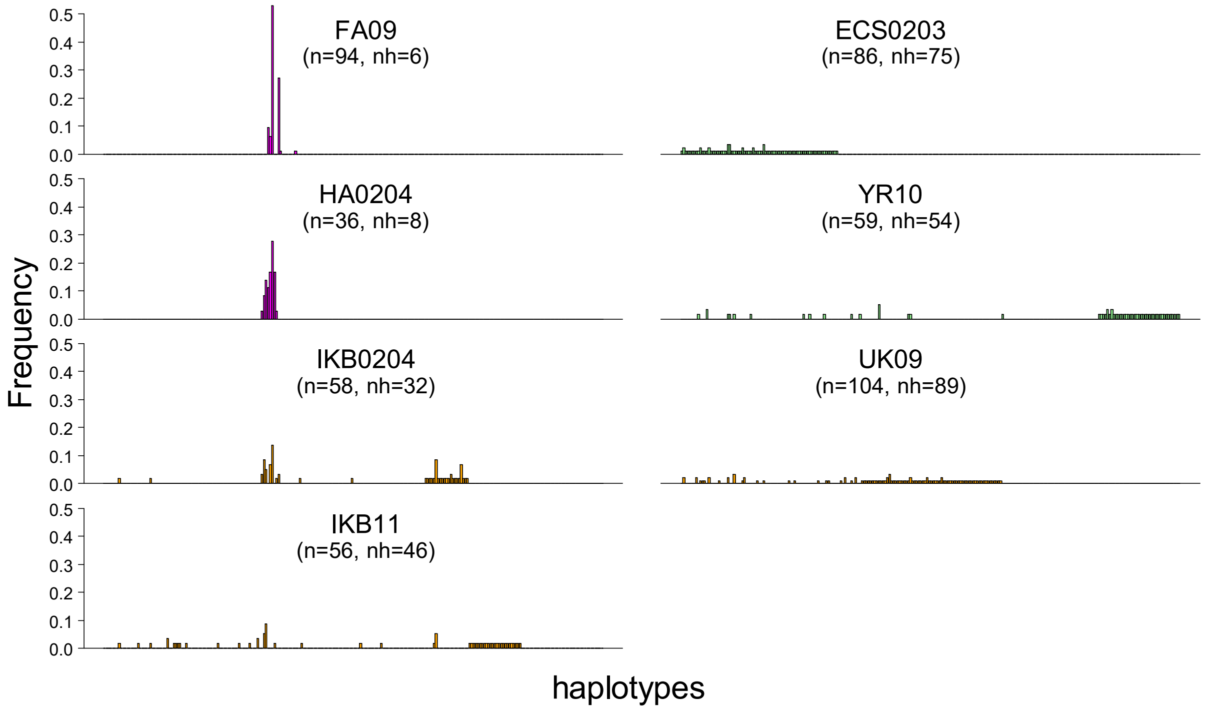

Supplementary Figure S1. Red sea bream mtDNA control region haplotype frequencies.

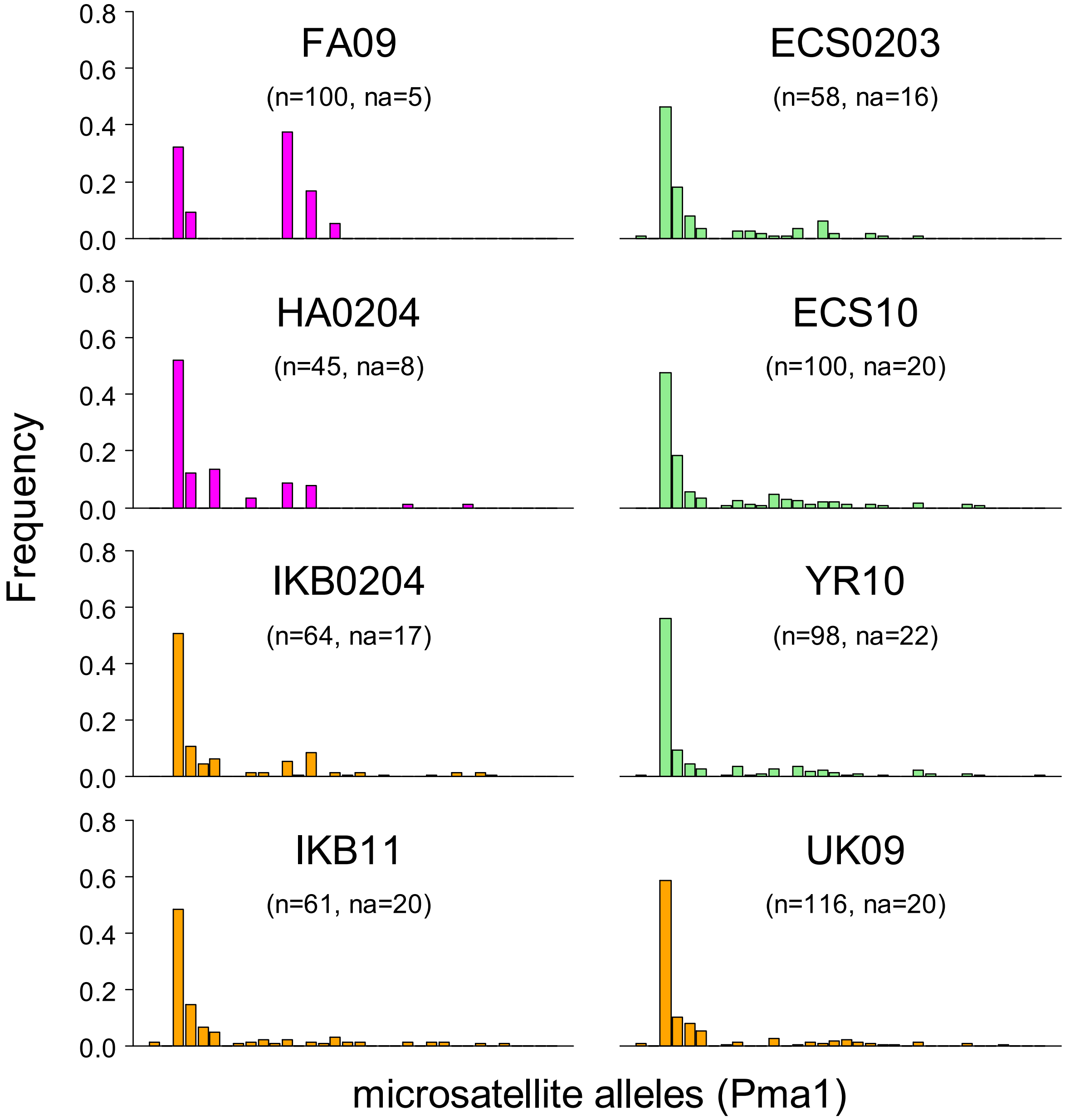

Supplementary Figure S2a. Red sea bream *Pma*1 locus allele frequencies.
n; sample size, na; number of alleles.

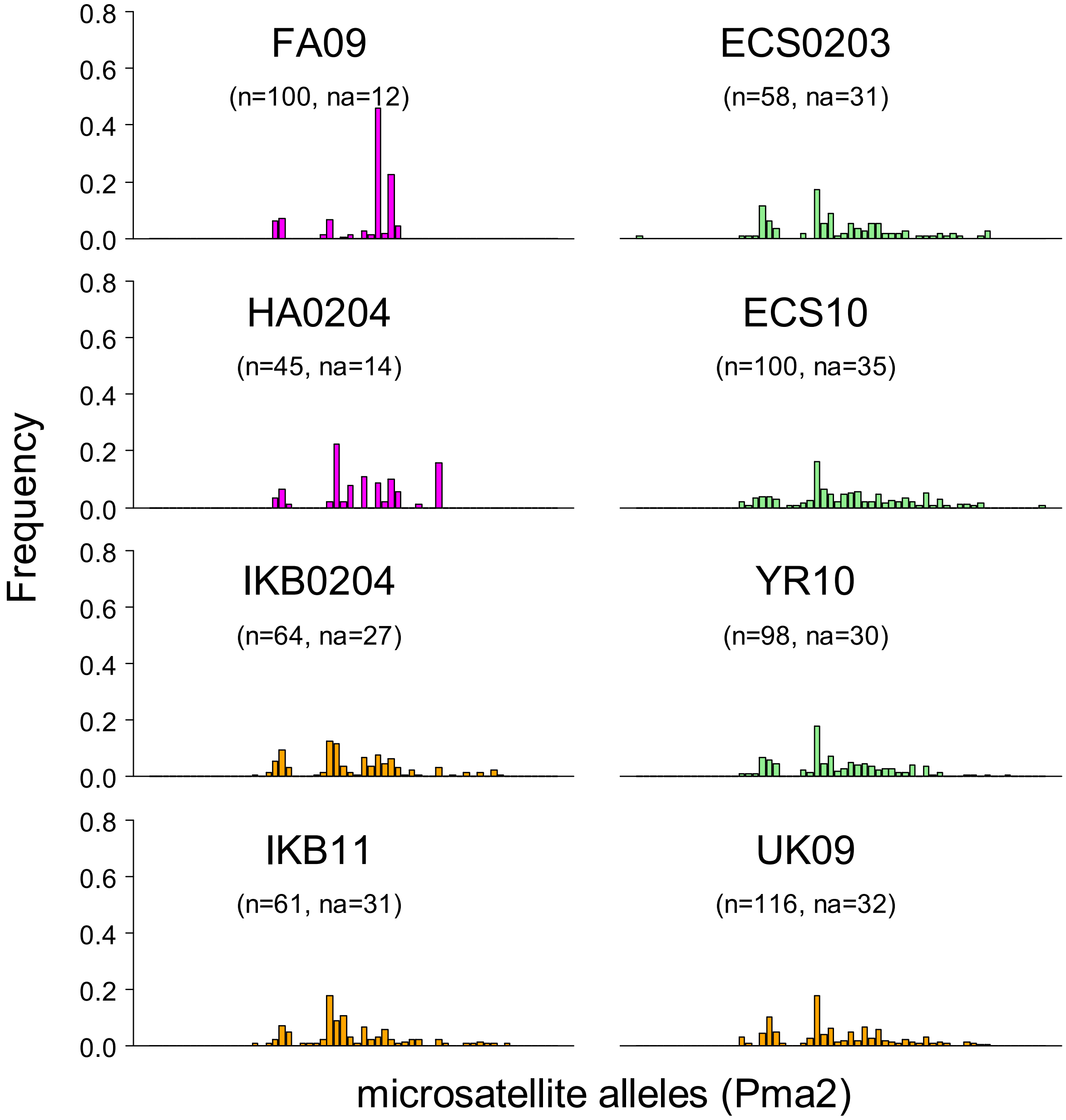

Supplementary Figure S2b. Red sea bream *Pma*2 locus allele frequencies.
n; sample size, na; number of alleles.

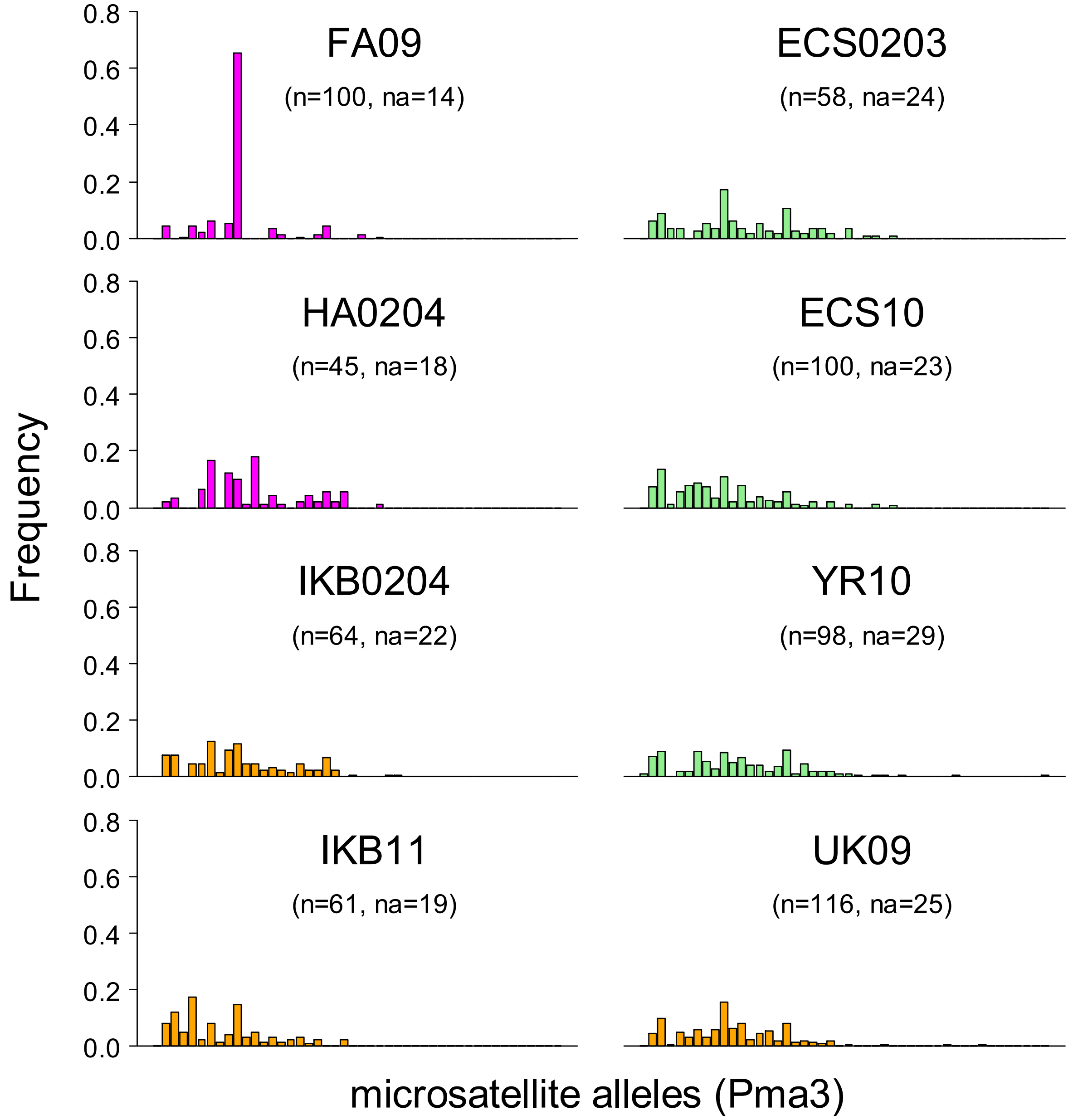

Supplementary Figure S2c. Red sea bream *Pma*3 locus allele frequencies.
n; sample size, na; number of alleles.

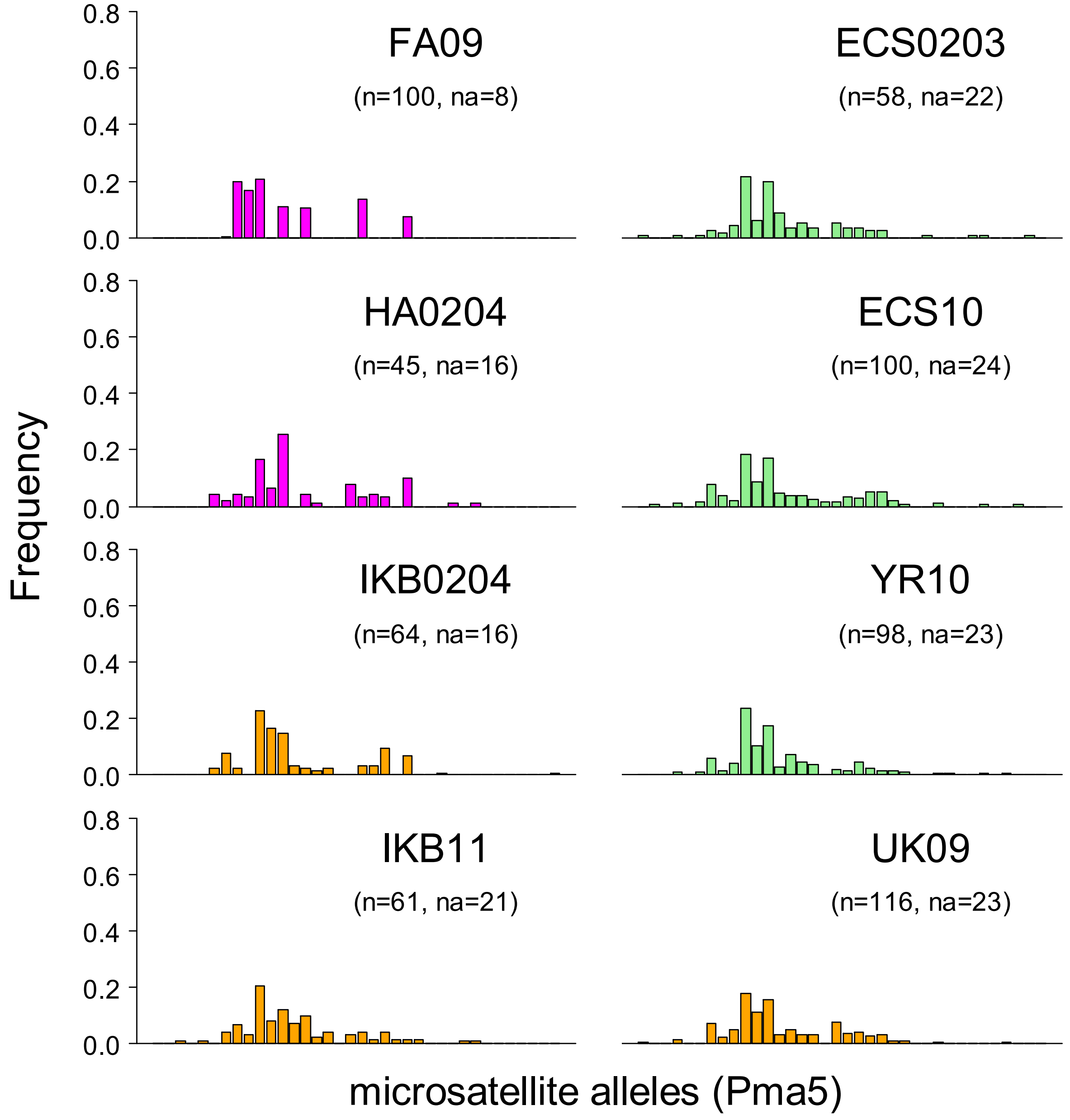

Supplementary Figure S2d. Red sea bream *Pma*5 locus allele frequencies.
n; sample size, na; number of alleles.

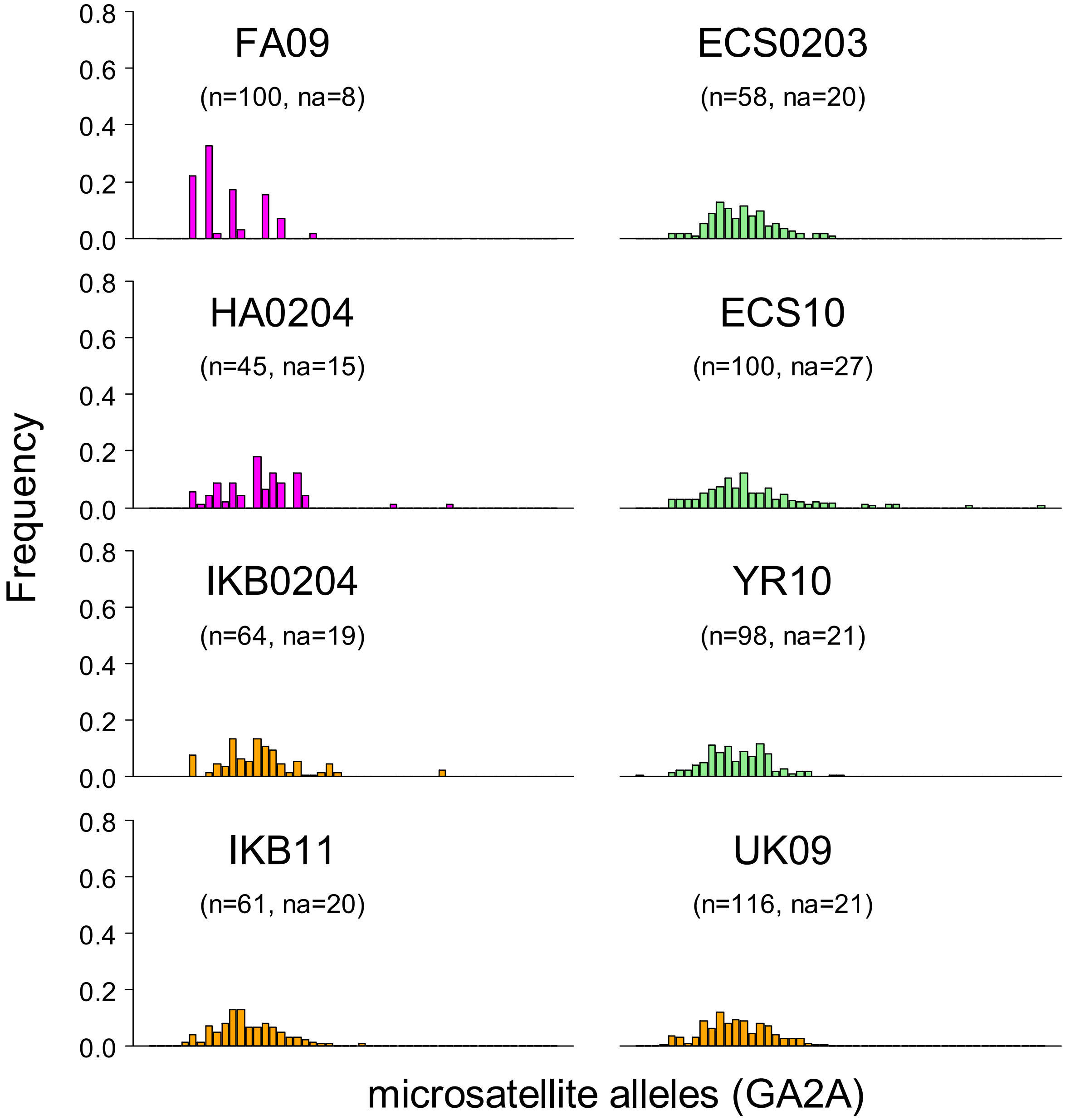

Supplementary Figure S2e. Red sea bream *GA2A* locus allele frequencies.
n; sample size, na; number of alleles.

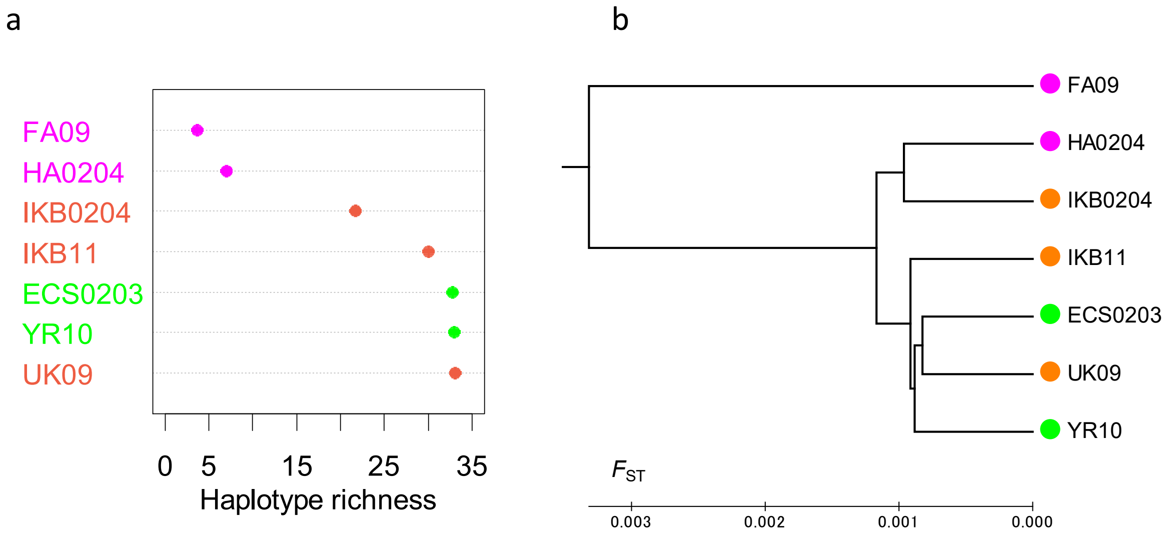

Supplementary Figure S3. Red sea bream genetic diversity and population structure for mitochondrial DNA control region. **a**, Haplotype richness. **b**, EBF_{ST} UPGMA dendrogram.

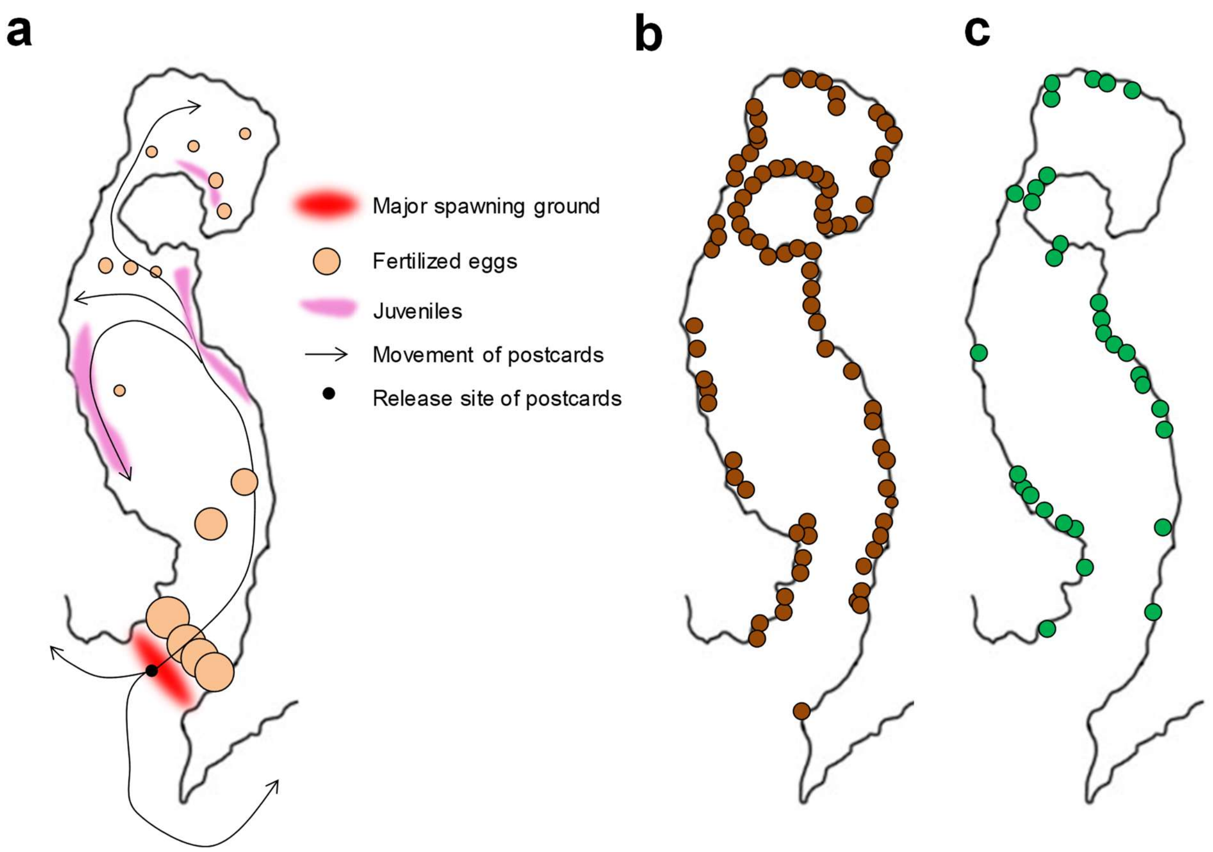

Supplementary Figure S4. Reproduction process of red sea bream and distribution of seaweeds communities in Kagoshima Bay. **a,** Major spawning grounds and distribution of fertilized eggs and juveniles (redrawn from the results of the field surveys conducted by KPFTDC, unpublished data). **b,** Hijiki (brown alga, *Sargassum fusiforme*) and **c,** *Zostea marina* in 2006 (redrawn from the figures of the original study of Tanaka et al.[28]).

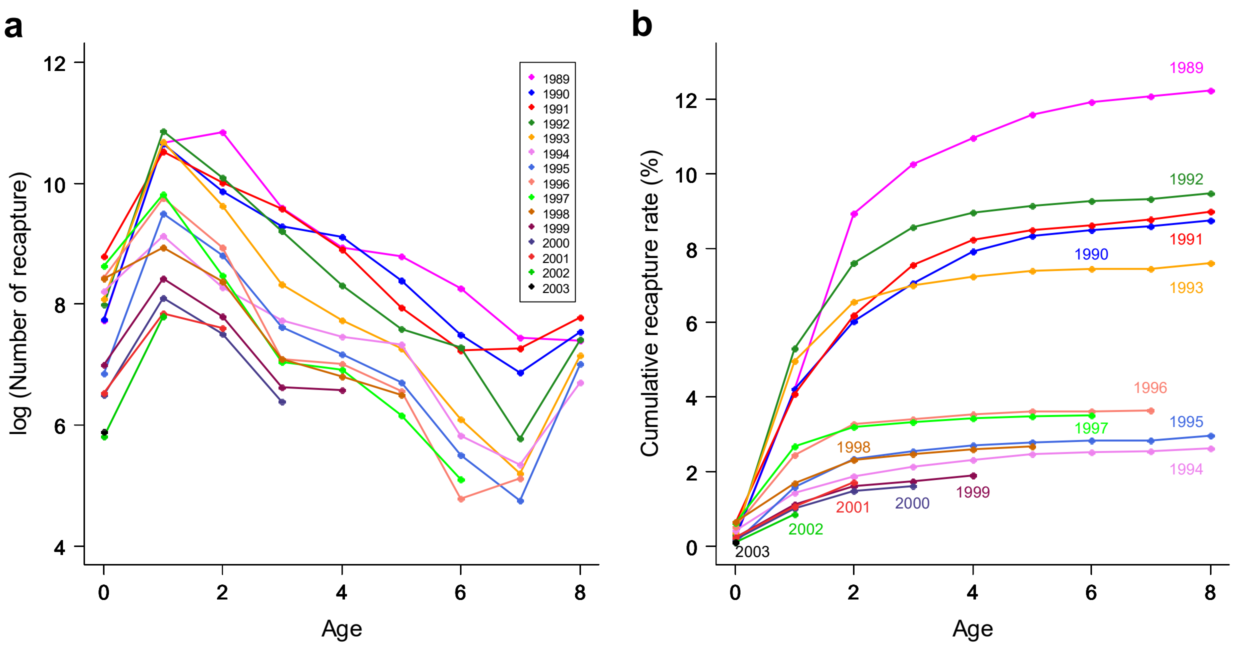

Supplementary Figure S5. Numbers of recapture (**a**) and cumulative recapture rates (**b**) of the red sea bream in Kagoshima Bay for each release year. Cumulative recapture rates for Age 1 and Age 8 (included older fish) were plotted on Fig. 1d. Redrawn from Shishidou et al.[29] (Data in Supplementary Data online).

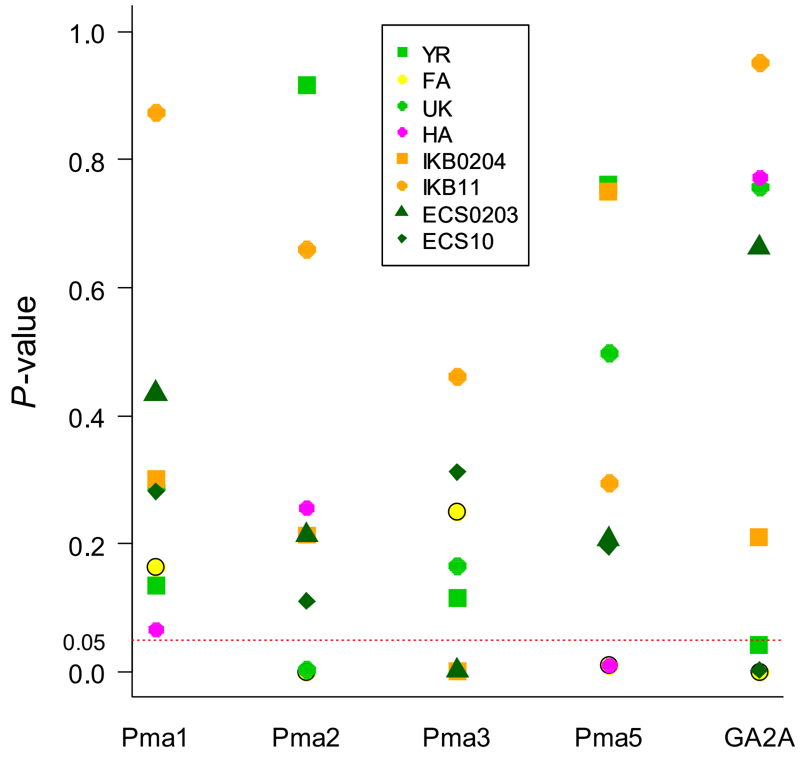

Supplementary Figure S6. Tests of Hardy–Weinberg equilibrium. Significance levels (*P*-values) at each locus for the red sea bream from each sampling location.
